# Delay in primordial germ cell migration in adamts9 knockout zebrafish

**DOI:** 10.1038/s41598-021-88024-x

**Published:** 2021-04-20

**Authors:** Jonathan J. Carver, Yuanfa He, Yong Zhu

**Affiliations:** 1 Department of Biology, Howell Science Complex, East Carolina University, 1000 E. 5th Street, Greenville, NC 27858 USA; 2 College of Fisheries, Guangdong Ocean University, Zhanjiang, Guangdong China

**Keywords:** Infertility, Developmental biology, Genetics, Molecular biology, Physiology

## Abstract

Adamts9 (a disintegrin and metalloproteinase with a thrombospondin type 1 motif, member 9) is one of a few metalloproteinases structurally conserved from *C. elegans* to humans and is indispensable in germ cell migration in invertebrates. However, *adamts9*′s roles in germ cell migration in vertebrates has not been examined. In the present study, we found zygotic expression of *adamts9* started around the germ ring stage and reached peak levels at 3 days post fertilization (dpf) in zebrafish. The migration of primordial germ cells (PGC) was completed within 24 hours (h) in wildtype siblings, while a delay in PGC migration was found at 15 and 24-h post-fertilization (hpf) in the Adamts9 knockout (KO). However, the delayed PGC migration in Adamts9 KO disappeared at 48 hpf. Our study suggests a conserved function of Adamts9 in germ cell migration among invertebrates and vertebrates. In addition, our results also suggest that Adamts9 is not essential for germ cell migration as reported in *C. elegans*, possibly due to expansion of Adamts family members and compensatory roles from other metalloproteinases in vertebrates. Further studies are required in order to elucidate the functions and mechanisms of metalloproteinases in germ cell migration and gonad formation in vertebrates.

## Introduction

Metalloproteinases serve essential roles in morphogenesis, tissue remodeling, and cell migration, all of which are important in normal or disease processes. From genome-wide association studies (GWAS), Adamts9 (a disintegrin and metalloproteinase with a thrombospondin type 1 motif, member 9) is associated with various human diseases, such as diabetes^1–3^, asthma^4^, arthritis^5,6^, artery calcification^7,8^, macular degeneration^9^, and cognitive aging^10^. However, Adamts9-dependent physiological processes, *adamts9* expression, and in vivo functions are still poorly understood mainly due to the lack of non-lethal vertebrate knockout animal models.

**Figure 1:**
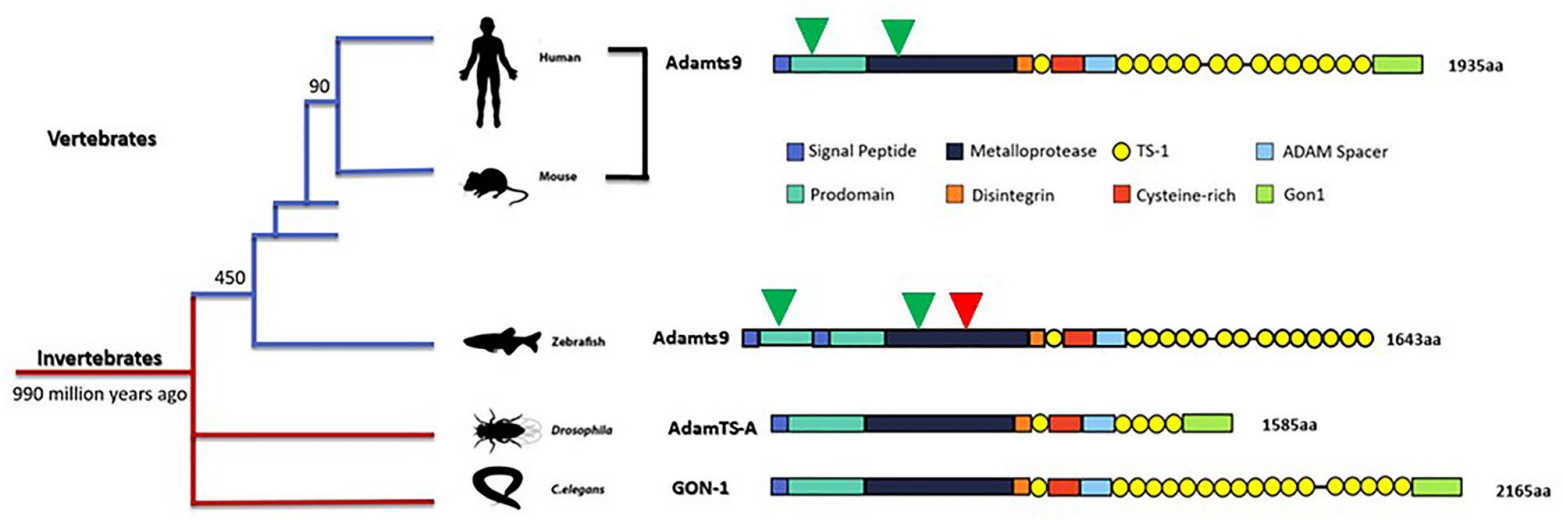
Conserved structure of Adamts9 from *C. elegans* to human. Different function domains are illustrated in different color. Green triangles show sites for two commercial antibodies (Triple Point Biologics, Inc., OR) generated against 180 peptide sequence of prodomain and metalloproteinase domain of human Adamts9, which share 41% and 86% sequence identity with zebrafish Adamts9, respectively. Red triangle shows CRISPR targeting site for generating zebrafish *adamts9* knockout. Two mutant zebrafish lines (∆10 and ∆11) which generated premature stop codons before the enzymatic active site (at 573aa) were selected and propagated^24^ (Please see supplemental Figs. 1 and 2, Supplemental Tables 1–4 for detail).

The formation of a functional gonad is essential for animals to reproduce. A functional, adult ovary or testis develops from a juvenile bipotential gonad via several physiological processes that include cell migration, apoptosis, proliferation, and tissue remodeling. These processes are regulated precisely by various cellular signaling molecules and proteinases. Adamts9 is one of few metalloproteinases structurally conserved from *C. elegans* to humans^11–13^ (Fig. 1), and it is required in gonad formation in invertebrates^14–16^. In the knockout of Adamts9 ortholog, *gon-1* in *C. elegans*, germ cells do not migrate, and the gonad develops as a disorganized mass of somatic and germ line tissues^14–16^. In *Drosophila,* the knockout of Adamts9 ortholog, AdamtTS-A causes the mis-migration of collective cells, including germ cells^17^. However, due to embryonic lethality in the knockout *Drosophila*^17^ and mouse models^18,19^, the functions and underlying mechanisms of Adamts9 during gonad development and formation are still unknown. It is also important to note that compared to invertebrates, members of the extracellular matrix (ECM) protein families and ADAMTS family expand dramatically in vertebrates^20^, which may lead to loss and gain of functions for Adamts9.

Our previous studies suggest that Adamts9 is critical for normal development of ovaries and ovulation in the zebrafish^21–24^. In the present study, we examined expression of Adamts9 in the embryonic development and its roles in germ cell migration. Zygotic expression of Adamts9 started around germ ring stage of embryos in zebrafish development. A delay in the migration of primordial germ cells was found during the gonadal development.

## Results

### Expression of Adamts9 in follicular cells and during embryonic development

Zebrafish Adamts9 (NP_001244125) shares high sequence identities with its orthologs in *C. elegans*, *Drosophila*, mice and human (Fig.1, supplemental Figs.1 and 2, supplemental Tables 1–4). The highest percentages of amino acid sequence identities (40-88%) between *C. elegans* and human were found in the metalloproteinase domain of Adamts9 (Supplemental Table 2), while an amino acid motif (HELGHXXXXXHDD) for the Adamts9 enzymatic activation site was completely conserved and aligned (Supplemental Fig. 1). Intriguingly, zebrafish Adamts9 has two propeptide domains, but lacks the GON-1 domain that is conserved from *C. elegans* to human. Currently, we have no evidence to support that zebrafish Adamts9 was truncated, or the unique GON-1 domain was lost in some vertebrate species (https://www.ncbi.nlm.nih.gov/gene/56999/ortholog/?scope=7776&term=ADAMTS9), even the duplication of propeptide domain might be unique to the zebrafish.

**Figure 2:**
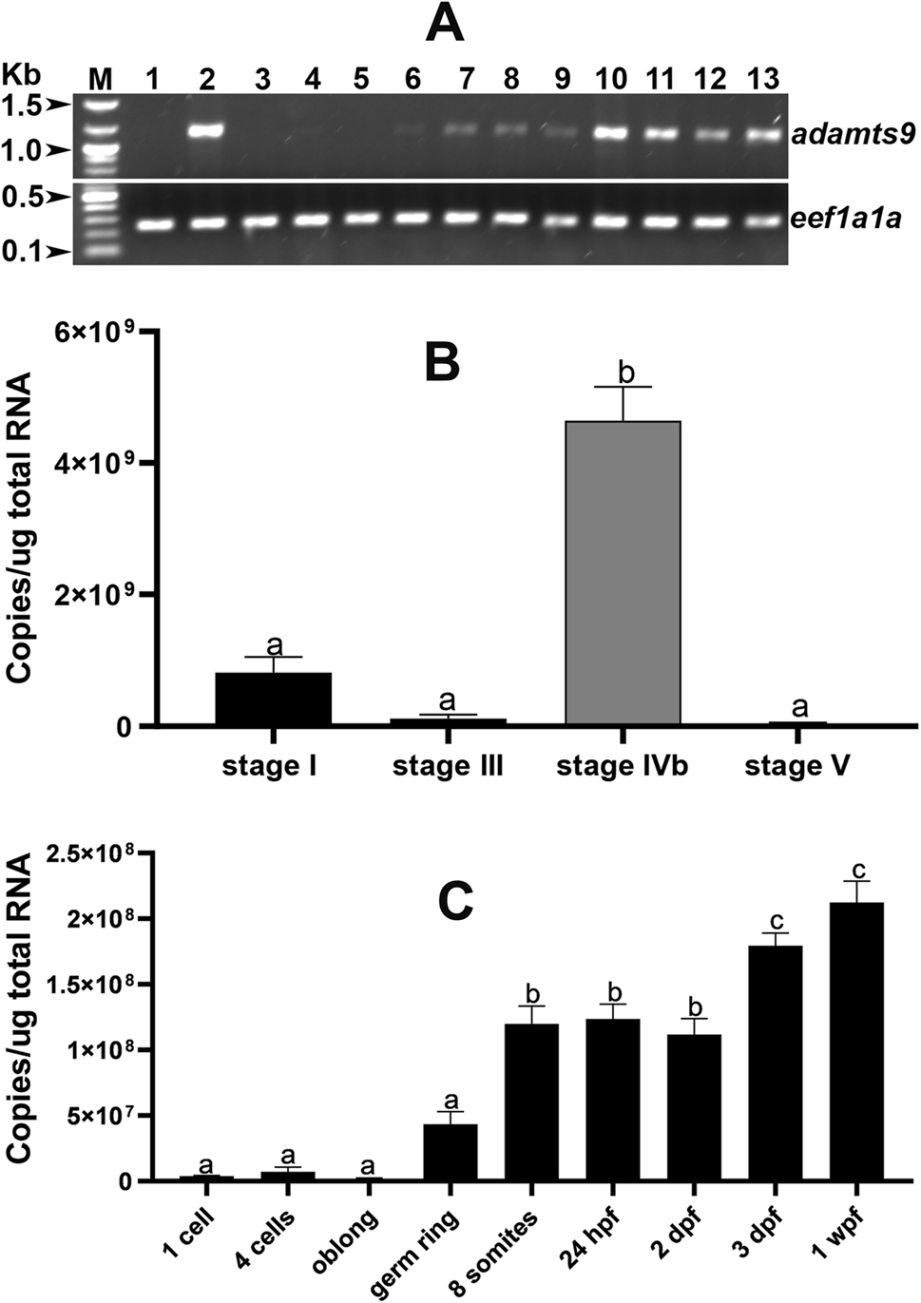
Strong *adamts9* expression in preovulatory follicular cells and developing embryos. (**A**) Expression of *adamts9* determined by RT-PCR. Top panel is *adamts9* transcript analyzed by RT-PCR (35 cycles), bottom panel is expression of a house keep gene, eukaryotic translation elongation factor 1 alpha 1a (*eef1a1a*). M. NEB 1 kb plus DNA ladder; 1. Ovary (fully grown immature follicles); 2. Preovulatory stage IVb follicles; 3. Ovulated stage V oocytes; 4. One cell stage embryos; 5. Four cell stage embryos; 6. Oblong stage embryos (~ 3.5 hpf, hours post fertilization); 7. Germ-ring stage embryos (~ 5.5 hpf); 8. Eight somite stage embryos (~ 11.5 hpf); 9. 24 hpf (hours post fertilization) embryos; 10. Two dpf (days post fertilization ) embryos; 11. Three dpf embryos; 12. Six dpf embryos; 13. Six wpf (weeks post fertilization) gonad. Please see Supplemental Figs. 3 and 4 for full agarose gel images. (**B**) Strong *adamts9* expression in preovulatory follicles, low or no expression in immature follicles or ovulated oocytes determined by real-time quantitative PCR (qPCR). Stage I and III immature follicles,  IVb preovulatory follicular cell enclosed oocytes and stage V ovulated oocytes were collected from mature AB wildtype females between 7–8:30 am (lights on 8:30am-10:30 pm). Results were presented as mean ± SEM (n = 5). Different letters above the error bars indicate that those groups are significantly different from each other at *p* < 0.05. (**C**) Zygotic expression of *adamts9* in developing embryos determined by qPCR. Results were presented as mean ± SEM (n = 6). Different letters above the error bars indicate that those groups are significantly different from each other at *p* < 0.05.

The expression of *adamts9* transcript was low in immature oocytes, but dramatically increased in the follicular cells of stage IVb mature and preovulatory ovarian follicles. The expression of *adamts9* decreased beneath the detection limit in stage V ovulated oocytes (Fig. 2A,B). These results suggest *adamts9* is expressed highly in somatic cells adjacent to mature germ cells, but not in the mature germ cells.

Zygotic expression of *adamts9*′s transcript was under the PCR detection limit between 1 cell and oblong stage of embryos (~ 4 h post fertilization (hpf)), gradually increased around germ ring stage (~ 6 hpf), and reached a peak level around 3 days post fertilization (dpf) (Fig.2A,C).

**Figure 3:**
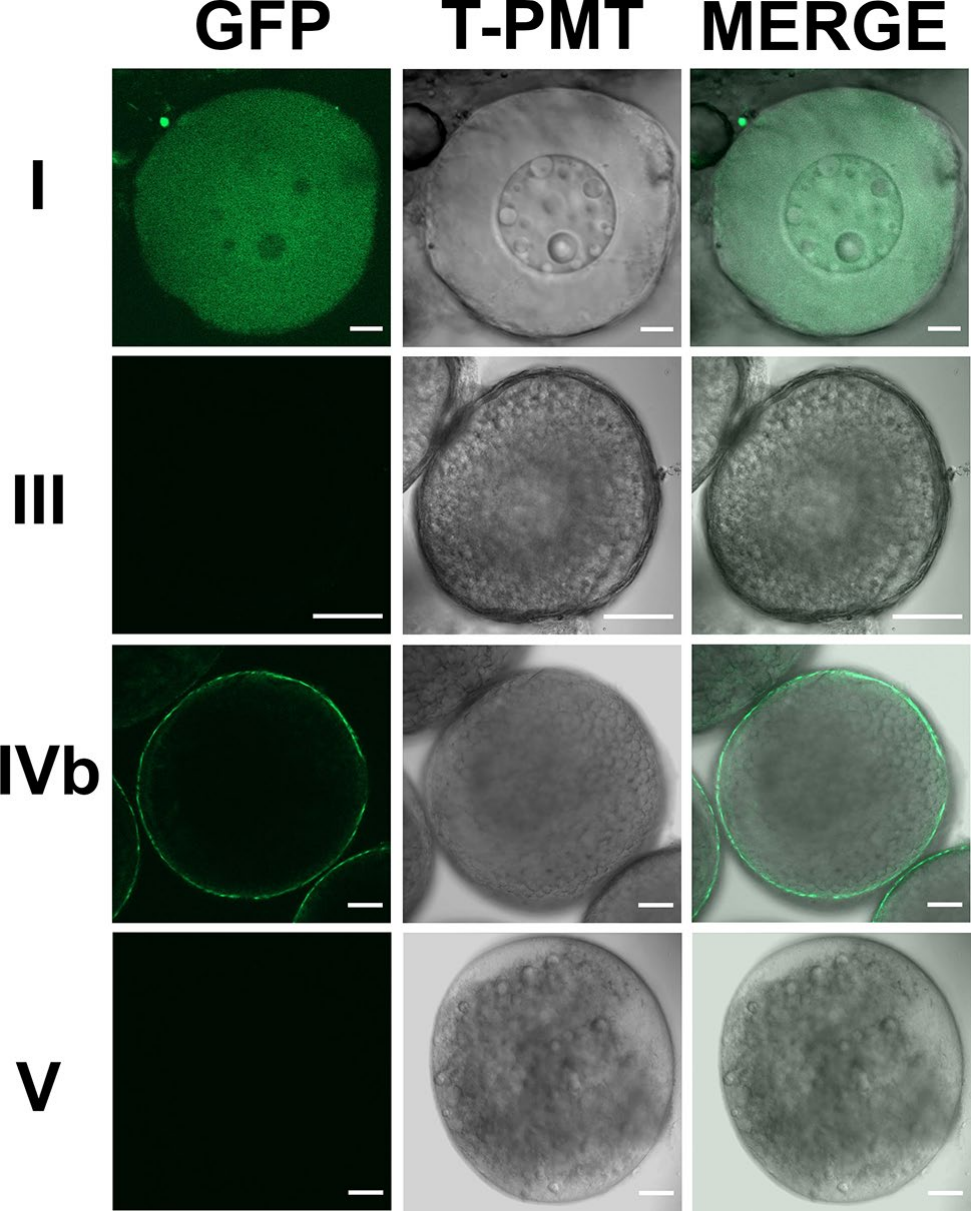
Strong expression of Adamts9 in preovulatory follicles, weak or no expression in immature ovarian follicles or ovulated oocytes in Adamts9 transgenic lines (*Tg(adamts9:GFP)*). The expression of Adamts9 was determined by EGFP expression driven by *adamts9* promoters located in a 4.5 kb upstream sequence of *adamts9* start codon. Representative confocal slice images of stage I (immature pre-vitellogenic), III (immature vitellogenic), IVb (mature & preovulatory) follicles and stage V ovulated oocytes were shown. Follicles or oocytes were confocal imaged under EGFP and transmit light (T-PMT) channels. Similar expression was confirmed in the follicles or oocytes of several F1 and F2 adult females from five independent transgenic lines. Scale bar: 10 μm (stage I); 100 μm (stages III, IVb, V).

**Figure 4:**
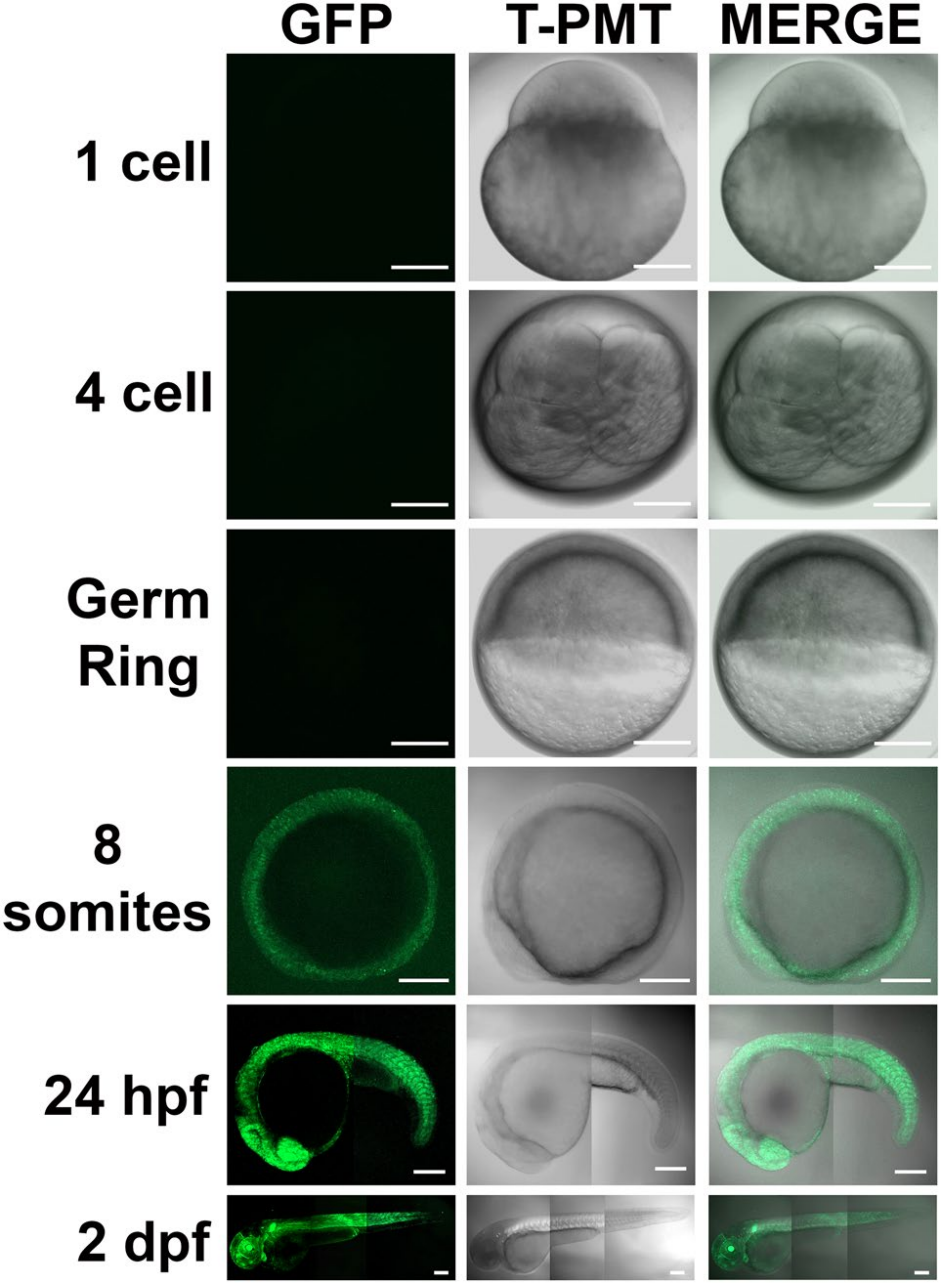
Expression of Adamts9 in F2 transgenic (*Tg(adamts9:GFP)*) embryos during embryonic development. The expression of Adamts9 was determined by EGFP expression driven by *adamts9* promoters located in a 4.5 kb upstream sequence of *adamts9* start codon. Representative confocal z-stack images of various stages of development embryos were imaged by a confocal microscope under EGFP or transmit light (T-PMT) channels. Similar expression was confirmed in multiple F2 embryos from five independent F1 transgenic lines (*Tg(adamts9:GFP)*). Scale bar: 200 μm.

**Figure 5:**
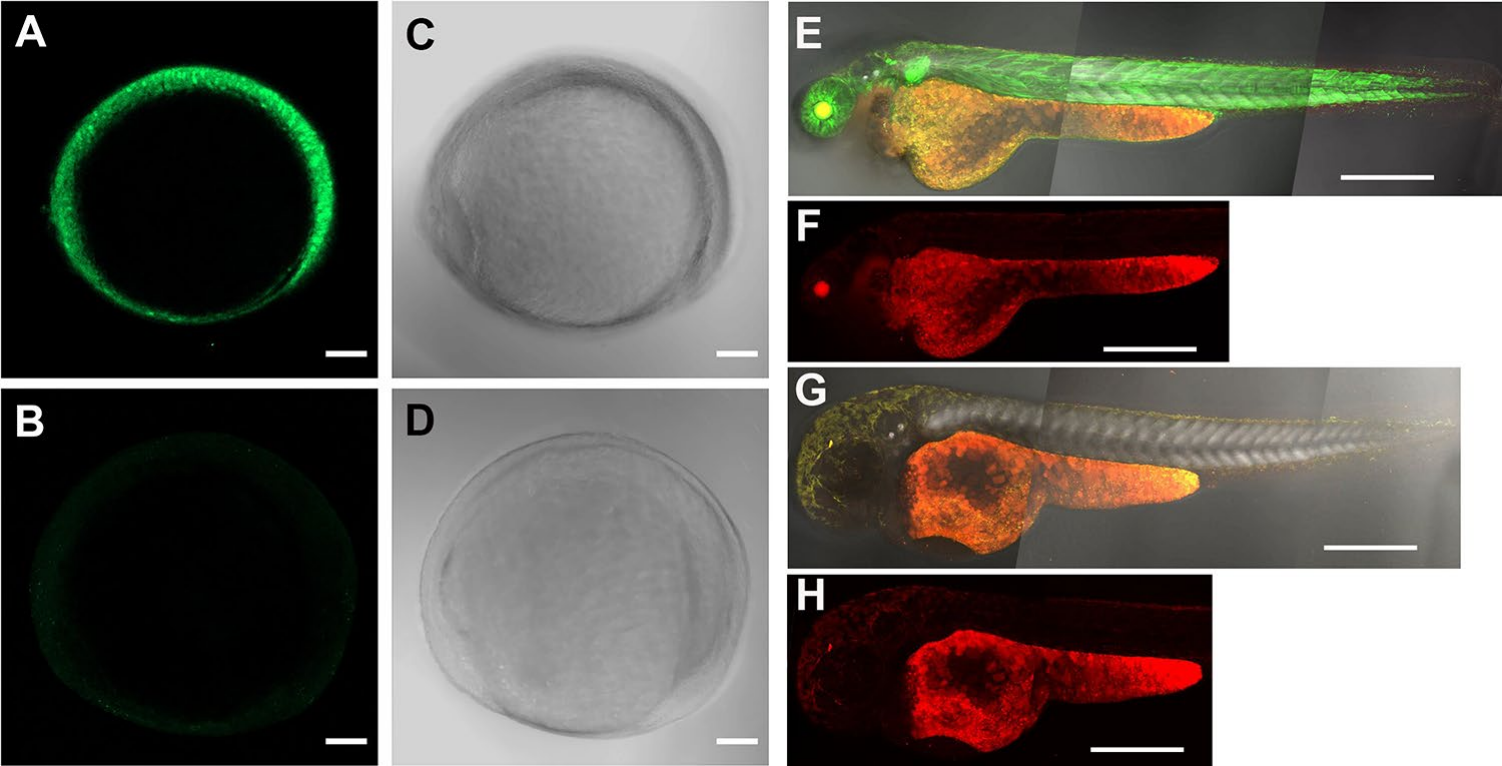
Strong Adamts9 expression in F2 transgenic (*Tg(adamts9:GFP)* zebrafish embryos. The expression of Adamts9 was determined by EGFP expression driven by *adamts9* promoters located in a 4.5 kb upstream sequence of *adamts9* start codon. Same conditions were used for imaging embryos under the EGFP, red (for monitoring autofluorescence), and transmit light (T-PMT) channels. Picture (**A**) is z-stack confocal images of a representative F2 transgenic embryo at 10 h post fertilization (hpf) from GFP channel; Picture B is a wildtype control embryo imaged under same condition. Pictures (**C**) or (**D**) are corresponding images of same embryo from pictures A or B using T-PMT channel. Pictures (**E**,**F**) are confocal z-stack images of a representative F2 transgenic embryo at 48 hpf, while pictures (**G**,**H**) are confocal z-stack images of a wildtype embryo at 48 hpf. Pictures (**E**,**G**) are merged confocal z-stack images from all three channels, while pictures (**F**,**H**) were confocal z-stack images from red channel to show background. Scale bar: 100 μm (**A**–**D**), 400 μm (**E**–**H**).

In corresponding to the expression of *adamts9* transcripts, strong GFP expression driven by *adamts9* promoters was observed in mature/preovulatory stage IVb follicles, while weak or no GFP expression was observed in immature follicles (stage I, III) or ovulated stage V oocytes. (Fig. 3). Zygotic expression of GFP was observed around bud stage of embryos, gradually increased and become obvious around 8 somite stages of embryos, and reached peak levels after 2 dpf (Figs. 4 and 5).

### Delay in primordial germ cell (PGC) migration in Adamts9 knockout (KO)

**Figure 6:**
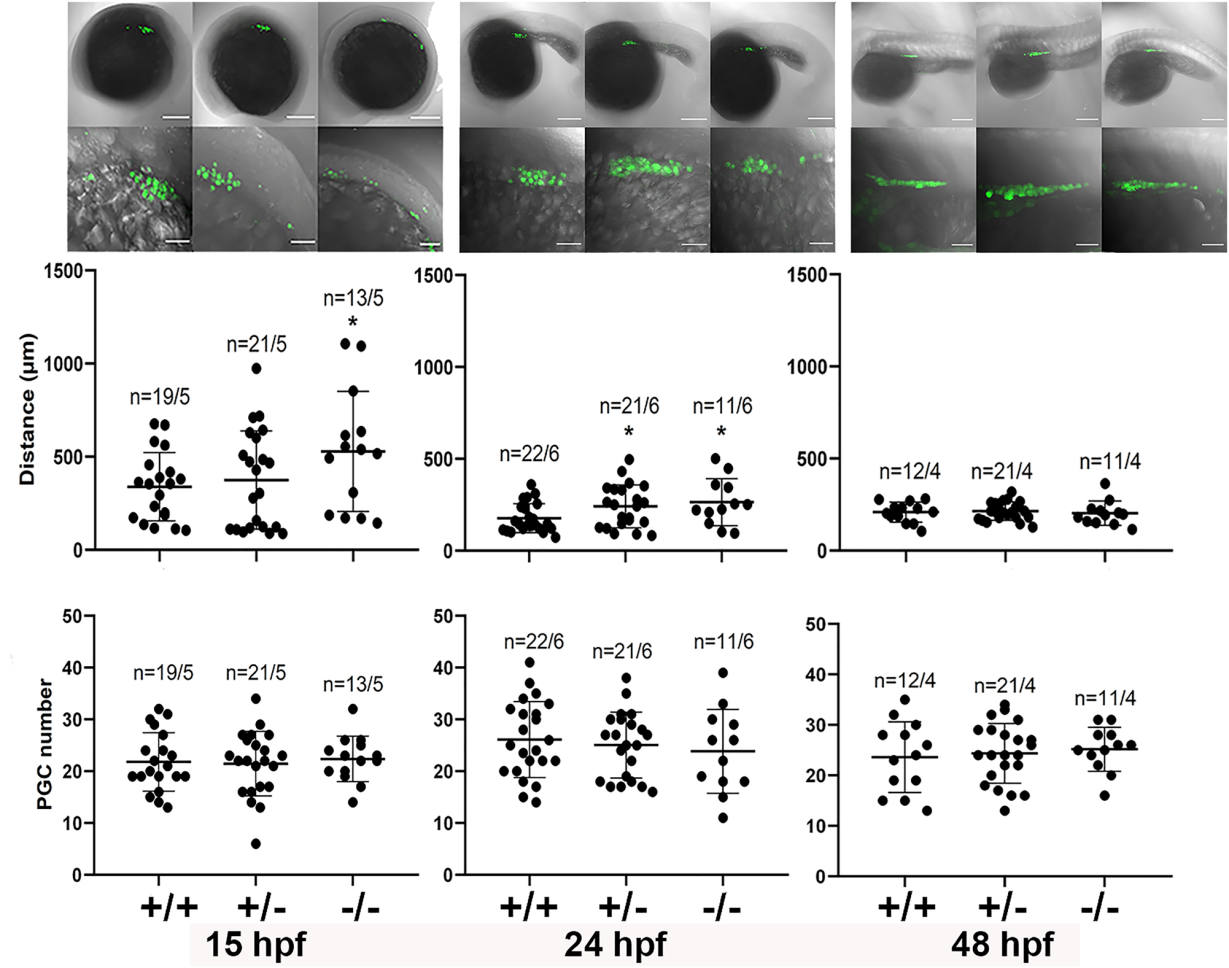
Delayed primordial germ cell (PGC) migration in Adamts9 knockout zebrafish during early development. PGC is labeled with GFP by crossing Adamts9 KO with *Tg(vasa:GFP).* Distance between two most distant PGCs were determined as an indicator of PGC migration (See Fig. 7 for detail). Showing distance, number of PGC, or representative images of zebrafish embryos at 15, 24 or 48 h post fertilization (hpf) in wildtype (+/+), heterozygous (+/–), and homozygous (–/–) Adamts9 KO. Embryos from at least 4 sets of parents were analyzed. The numbers on the left side of the forward slash is the number of embryos analyzed, and the numbers on the right side of the forward slash indicate sets of parents used for producing these embryos. Top panels are representative confocal images showing entire or part of embryos with GFP labeled PGC at a low magnification (scale bar: 200 μm). Bottom row are magnified confocal images of the embryos from the top (scale bar: 50 μm).

We generated a zebrafish line with all the germ cells labeled with GFP in Adamts9 KO background (*adamts9*^+*/−*^; *Tg(vasa:GFP)*). We crossed these heterozygotes and obtained wildtype (*adamts9*^+/+^), heterozygotes (*adamts9*^+*/−*^), and homozygotes (*adamts9*^−/−^) sibling. PGC completed their migration and tightly clustered together at 24hpf in wildtype (*adamts9*^+/+^) fish. We found a delay in PGC migration in the Adamts9 KO (*adamts9*^−/−^) zebrafish. PGCs spread in wider area in homozygotes than those in wildtype sibling at 15 and 24 hpf, i.e. delay in the migration of PGCs (Fig. 6). However, this delayed migration effect disappeared at 48 hpf (Fig. 6). In addition, all the PGCs migrated to the gonadal ridge despite the delay of germ cell migration in Adamts9 KO zebrafish between 15 and 24 hpf. There is no significant difference in the numbers of germ cells among different genotypes (Fig. 6).

## Discussion

**Figure 7:**
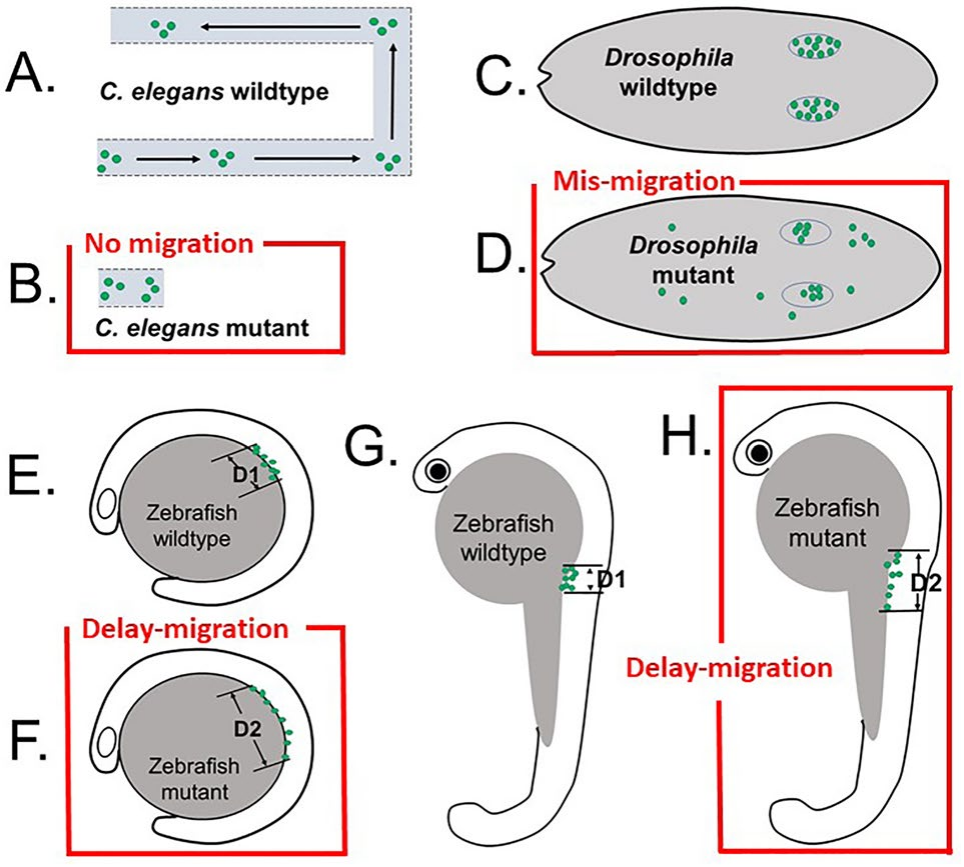
Schematic drawings show difference in the defects of germ cell migration in Adamts9 or its ortholog knockouts in *C. elegans, Drosophila*, and zebrafish models. Green dots denote primordial germ cells (PGC). Only the posterior gonad arm is shown for *C. elegans*. (**A**) PGCs migrate in wildtype *C elegans*, (**B**) no migration occurs in *gon-1* mutant (modified from Blelloch and Kimble^14,15^). (**C**) PGCs migrate correctly in wildtype *Drosophila* and clustered together at gonadal ridge at stage 16 embryo. (**D**) PGC mis-migrate in AdamTS-A knockout embryos (modified from Ismat et al.^17^). E–H. All PGC migrate toward gonadal ridge in zebrafish embryos, though these PGC were distributed in wider area (D2 > D1) in the Adamts9 knockouts at 15 h post fertilization (hpf, **E**: wildtype; **F**: Adamts9 KO) and 24 hpf (**G**: wildtype; **H**: Adamts9 KO), i.e. delayed migration. The migration of PGC is completed around 24hpf in wildtype embryos, but 48hpf in Adamts9 KO zebrafish embryos (see Fig. 6 for detail).

Matrix metalloproteinases (MMPs) are well known for their involvements in cell motility such as stem cell migration or cancer cell invasion^30–34^. ADAMTS is a subgroup of secreted zinc metalloproteases with several distinct domains separated from classical MMPs^35,36^. Studies of Adamts9 orthologs in *C. elegans* and *Drosophila* suggest this proteinase may release a signal or clear a path in order for PGC to migrate appropriately^14–17^ in invertebrates. However, studies of possible involvements of metalloproteinases in PGC migration is still unknown in vertebrates^37^. In present study, we provided first evidence that a metalloproteinase, Adamts9, plays a role in the PGC migration in a vertebrate model. The availability of several transgenic lines that label PGCs with fluorescent proteins and nearly transparent embryos made zebrafish an excellent choice for studying germ cell migration at a high resolution within a live organism^38,39^. Transgenic zebrafish lines in a different genetic background, including Adamts9 KO allowed studying the roles of Adamts9 in PGC development starting from the earliest stages of their development. We determined the expression of Adamts9 in early development. We also determined germ cell migration and numbers of germ cells in the homozygous Adamts9 KO in comparison to their wildtype and heterozygous siblings. Our results suggest a conserved function of Adamts9 in germ cell migration among vertebrates and invertebrates (Fig. 7).

Our results also suggest that Adatms9 is not essential in the germ cell migration as demonstrated in *C. elegans*^14–16^ (Fig. 7). It should be noted that zebrafish Adamts9 lacks GON-1 domain. The role of Adamts9 in germ cell migration may be shared by other proteinases due to loss of a function domain in zebrafish. However, studies of Adamts9 ortholog (AdamTS-A) in *Drosophila* also suggest that this enzyme is not essential for germ cell migration as some germ cells migrate appropriately, while others mis-migrate^17^. None of previous studies have reported survival or number of germs in the knockouts. In present study, we found the numbers of PGCs were not affected in Adamts9 KO zebrafish embryos.

One single gene, the ancestor of Adamts, is found in the sponge corresponding to the origin of multi-cellularity and embryogenesis^20^. Up to 8 Adamts members are found in invertebrates. The Adamts family expanded dramatically during Metazoan evolution to 19 genes in vertebrates including zebrafish, mice, and humans^20,35,36^. The expansion of Adamts members in vertebrates may lead to gain and loss functions for each Adamts family member, which could explain diminished roles of Adamts9 in germ cell migration in vertebrates. On the other hand, heterozygotes (*adamts9*^+*/−*^) was used to produce wildtype (+/+), heterozygote (+/−) and homozygote (−/−) embryos due to female infertility in homozygous KO. Therefore, we could not completely exclude the possibility that maternally deposited Adamts9 from heterozygous females may have exerted influence on PGC migration. Future studies, such as using conditional knockouts, will exclude such possibilities. However, the expressions of *adamts9*  in wildtype were under the detection PCR limit in both ovulated oocytes and in early embryos from 1 cell stage to oblong stage. Zygotic expression of Adamts9 began around germ ring stage of embryos (~ 6 hpf, He et al., in review). Delayed germ cell migration observed between 15 and 24 hpf happened after zygotic expression of Adamts9 in zebrafish embryos, suggesting little or no role of maternal deposited Adamts9 if present in zebrafish.

The expression of *adamts9* orthologs (GON-1 in *C. elegans* and AdamTS-A) was found in somatic cells adjacent to germ cells, but not in the germ cells^14–17^. Interestingly, ectopic expression of GON-1 or AdamtTS-A in the germ cells in *C. elegans* or *Drosophila* would rescue germ cell migration defects. Adamts9 is expressed in various tissues including ovaries and testes in human and mice according to NIH gene database; however, whether Adamts9 is expressed in the somatic cells or germ cells of the gonads in human or mice is still unknown. Interestingly, we found *adamts9* is expressed in early germ cells during early development or in adult gonads, although the expression was low. It is possible that the low expression of *adamts9* in the germ cells was missed in previous studies in *C. elegans* and *Drosophila*. Alternatively, the low expression in the germ cells found in zebrafish is related to the dramatic expansion of Adamts members, and thereafter gain of functions in the germ cells in vertebrates. Further studies in other vertebrate species are required to confirm this hypothesis. Intriguingly, *adamts9* expression decreased as follicles grow, disappeared in the germ cells then increased dramatically in adjacent follicular cells in preovulatory follicles (stage IVb, Figs. 2 and 3). The dramatic increase of *adamts9* in the follicular cells of preovulatory follicles is due to increased LH and progestin signaling to prepare these follicles for ovulation^21–24^. However, the regulatory mechanisms, the cause and the effects of *adamts9* decrease in growing follicles are still unknown.

Adamts9 knockout in mice is lethal to embryos giving evidence of its essential role during development. It is highly expressed in the mouse genital tubercle and ovary^40^. However, due to embryonic lethality in the knockout *Drosophila* and mouse models^17–19^, the specific functions and underlying mechanisms of Adamts9 during gonad development, differentiation, and maintenance are still unknown. We generated zebrafish knockouts by targeting a CRISPR site prior to the enzymatic active site of Adamts9 (Supplemental Fig. 1)^24^. No immune-positive signals were detected in the follicular cells of zebrafish Adamts9 knockout, whereas strong positive signals were detected in the follicular cells of wildtype and heterozygous sibling^24^. Any residual of truncated proteins of Adamts9, if present, would have no enzymatic activities as it lost its active metalloproteinase sites in our zebrafish knockouts^24^.

It is well established that the number of germ cells is important for gonadal development and sex determination in zebrafish^41–43^. A threshold number of germ cells is required for ovarian development. The depletion of germ cells in development or the adult will lead to the development of males^41–43^. We hypothesized that the unusual development of ovaries and male biased sexual ratio found in Adamts9 KO was due to a defect in germ cell migration, early germ cell survival, and/or proliferation of PGCs. Our results show that removing Adamts9 had no effects in the numbers of germ cells during early development (15–48 hpf). Further studies are required to elucidate the processes and mechanisms of Adamts9 at late time points that could lead to male biased sex ratio, abnormal ovary, and female infertility reported in Adamts9 KO^24^.

## Materials and methods

### Animals

The AB strain of zebrafish (*Danio rerio*) used here originated from the Zebrafish International Resource Center and then propagated in our lab following previously published guidelines^25^. All methods were carried out in accordance with relevant guidelines and regulations. The study was carried out in compliance with the ARRIVE guidelines. All experimental protocols have been approved by the Institutional Animal Care and Use Committee (IACUC) at East Carolina University.

### Collection of ovarian follicles and embryos and extraction of total RNA

Various stages of AB wildtype embryos were collected at different times of development. Ovarian follicles were divided into different stages according to follicular size and morphological criteria^26^ with a slight modification^21–23^. Stage I, III, IVb and V ovarian follicles were collected from 4-month old mature female AB wildtype fish between 7:00am and 8:30am (lights on photoperiod 8:30am-10:30 pm). Samples were placed in 1.7 mL RNase-free microcentrifuge tubes (GeneMate) containing 200 μl RNAzol (Molecular Research Center, Inc., OH. Catalog: RN 190) and homogenized immediately. Total RNA was extracted from homogenized solutions according to the manufacturer’s protocol. For each sample, cDNAs was synthesized using 2 μg total RNA and a high-capacity cDNA Reverse Transcription kit (Thermo Fisher Scientific, Waltham, Massachusetts, USA, Catalog#4368814) following the manufacturer’s instructions.

### RT-PCR amplification of *adamts9*

A set of PCR primer (forward: 5′-GCGGTACGCGTGGTAAAATC-3′; reverse: 5′-AGGCATGTGGACATAACGCA-3′) targeting 1181 bp of 3′-UTR of *adamts9* was used for RT-PCR amplification. PCR amplification was carried out using a Taq DNA polymerase (New England Biolabs, Ipswich, Massachusetts, USA, Catalog#0273) with initial denaturation at 95 °C for 2 min followed by 35 cycles of 30 s denaturation at 95 °C, 30 s annealing at 65 °C, and 60 s elongation at 68 °C. Zebrafish eukaryotic translation elongation factor 1 alpha 1a (*eef1a1a*) showed stable expression in different stages of embryos and ovarian follicles, therefore was used as a housekeeping gene control. A set of PCR primers targeting 242 bp of coding region of *eef1a1a* (forward: 5′-AGTGTTGCCTTCGTCCCAAT-3′; Reverse:5′-CACACGACCCACAGGTACAG-3′) was used for PCR amplification. The efficiency of the PCR and authentic PCR products was confirmed by gel electrophoresis analysis. The PCR products were also cloned into pGEM-T easy vector and confirmed by Sanger sequencing. The concentrations of these plasmids were quantified on Nanodrop 2000 (Thermo Fisher Scientific, Waltham, Massachusetts, USA), serially diluted and used as DNA templates for generating standard curves described in the following paragraph.

### Real-time quantitative PCR (qPCR) amplification of *adamts9*

The levels of *adamts9* transcripts were also determined by quantitative real-time PCR (qPCR) using SYBR green dye (Invitrogen) and a CFX Connect real-time thermal cycler (Bio-Rad Laboratories, Hercules, California, USA). The qPCR reaction was conducted with initial denaturation at 95 °C for 3 min, followed by 45 cycles of 30 s denaturation at 95 °C, 30 s annealing at 65 °C, and 30 s extension at 72 °C using the specific primers (Forward: 5′-CTGTCTGCGCGGTGATTCTA-3′; Reverse: 5′-CTCTTGCAGGGGCGTGATTA-3′) and GoTaq G2 DNA polymerase (Promega, Madison, Wisconsin, USA). Each PCR mixture (15 μl) consisted of 7.795 μl DNase free water, 3 μl 5XGoTaq buffer, 1.5 μl 25 mM MgCl2, 0.3 μl 10 mM dNTP mix, 0.15 ml 10μM forward or reverse primer, 2 μl 5X diluted cDNA, 0.03 μl 100X SYBR green dye (final concentration 0.2X), and 0.075 μl Taq. The transcript levels, expressed as absolute values (copies/μg total RNA), were determined using Ct values of samples and a standard curve generated from serial known concentrations of plasmid containing the target region of *adamts9*. The efficiency of the PCR and authentic PCR products was further confirmed by analyses of melting curve, gel electrophoresis, and Sanger sequencing.

### Adamts9 expression analyzed by *adamts9* promoter driven EGFP

Detailed generation and characterization of EGFP expression driven by *adamts9* promoters in zebrafish will be reported in a separate manuscript. Briefly, a 4.5-kb upstream of *adamts9* start codon was cloned into pGEM-T easy vector (Promega), Sanger sequence confirmed, and then subcloned into a p5E-mcs entry vector. Multisite Gateway cloning (Invitrogen) was conducted with a 5′ entry vector containing 4.5 kb *adamts9* promoter sequence, a middle entry vector containing EGFP, a 3′entry vector with stop poly A signal, and a destination vector that expresses a GFP selection marker specifically in the lens of eye^27^. In this final vector, the expression of EGFP is controlled by *adamts9* promoters. Transgenic embryos with the insertion could be easily selected by the eye marker that displays green fluorescence at 48 hpf under a fluorescent dissecting microscope. About 500 nl mixture containing 20 ng/μl construct, 20 ng/μl Tol2 transposon, and phenol red indicator were microinjected into one cell stage of embryos. Multiple F0 transgenic embryos were selected based on the lens selection marker, raised to adults, crossed with AB wildtype, then produced several F1 lines. Subsequently, we established multiple F2 stable transgenic lines (*Tg(adamts9:GFP)*) with stable expression of EGFP in the zebrafish.

### Confocal imaging and analyses of GFP expression, migration and numbers of PGCs in Adamts9 knockout

Fresh, live follicles collected from adult transgenic females (*Tg(adamts9:EGFP)*) were placed immediately in 15 mM HEPS buffer (pH 7.8) containing 50% L-15 medium (Fisher Scientific, #41-300-039). Follicles were pipetted up and down several times to separate individual follicles. Then, individual follicles of various developmental stages were placed on a depression glass slide and mounted in 1.2% low melting point agarose and immediately imaged by confocal microscopy.

Live transgenic embryos of various developmental stages were collected by crossing male transgenic fish (*Tg(adamts9:EGFP)*) with AB females. Individual embryos were placed on a depression glass slide, mounted in 1.2% low melting point agarose, and immediately imaged by confocal microscopy. Five independent F1 transgenic lines were used to confirm similar expression among all the transgenic lines.

Adamts9 knockout fish were generated and reported in our previous study^24^. Vasa is an RNA helicase expressed exclusively in primordial germ cells (PGCs)^28^. By using vasa promoter to drive EGFP expression, it is possible to visualize PGCs in zebrafish embryos with a laser confocal scanning microscope. Adamts9 knockout male fish (*adamts9*^−/−^) were crossed with *Tg(vasa:EGFP)*^29^. Embryos were collected, raised to adult, genotyped, and then in-crossed to obtain a transgenic line with all germ cells labeled with EGFP in Adamts9 knockout background (*adamts9*^+*/−*^; *Tg(vasa:GFP)*). This transgenic line was used for generating wildtype (+/+), heterozygote (+/–) and homozygote (−/−) embryos for confocal imaging of PGCs.

Zebrafish embryos were collected at 15, 24, 48 hpf, fixed in 10% buffered formalin for four hours at room temperature. Embryos were subsequently washed with distilled water, a PBS solution, and then increasing concentrations of methanol, before storage in 100% methanol at − 20 °C until analyses. Individual embryos were mounted onto a depression glass slide in 1.2% low melting point agarose and then imaged by confocal microscopy. Distance between two most distant PGCs was determined as an indicator of PGC migration using Zen 2.6 software. The numbers of germ cells were determined with aid of computer software (Imaris, Bitplane Inc, Zürich, Switzerland).

### Statistical analysis

All results were presented as mean ± SEM. Two-sample two tailed t-test was used to analyze the effect of genotypes on germ cell migration, One-way ANOVA was used to analyze the gene expression. Statistical significance was set at *p* < 0.05. All Statistical analysis were conducted using GraphPad Prism.

## Supplementary Information

Supplementary Information
