# Publisher Correction: Delay in primordial germ cell migration in adamts9 knockout zebrafish

**DOI:** 10.1038/s41598-021-89717-z

**Published:** 2021-05-12

**Authors:** Jonathan J. Carver, Yuanfa He, Yong Zhu

**Affiliations:** 1 Department of Biology, Howell Science Complex, East Carolina University, 1000 E. 5th Street, Greenville, NC 27858 USA; 2 College of Fisheries, Guangdong Ocean University, Zhanjiang, Guangdong China

Correction to: *Scientific Reports* 10.1038/s41598-021-88024-x, published online 20 April 2021

The original version of this Article contained errors in the in-text citations.

Firstly, in the Materials and methods section under the subheading ‘Confocal imaging and analyses of GFP expression, migration and numbers of PGCs in Adamts9 knockout’,

“Adamts9 knockout male fish (*adamts9*^−/−^) were crossed with *Tg(vasa:EGFP)*^30^”

now reads:

“Adamts9 knockout male fish (*adamts9*^−/−^) were crossed with *Tg(vasa:EGFP)*^29^”

Additionally, in the Discussion section,

“Matrix metalloproteinases (MMPs) are well known for their involvements in cell motility such as stem cell migration or cancer cell invasion^29,30,31,32,33,34^”

now reads:

“Matrix metalloproteinases (MMPs) are well known for their involvements in cell motility such as stem cell migration or cancer cell invasion^30–34^”

Also in the Discussion section,

“It is well established that the number of germ cells is important for gonadal development and sex determination in zebrafish^40,41,43^. A threshold number of germ cells is required for ovarian development. The depletion of germ cells in development or the adult will lead to the development of males^40,41,42,43^”

now reads:

“It is well established that the number of germ cells is important for gonadal development and sex determination in zebrafish^41,42,43^. A threshold number of germ cells is required for ovarian development. The depletion of germ cells in development or the adult will lead to the development of males^41,42,43^”

These errors have now been corrected in the HTML and PDF versions of the Article.

