# Supplementary material for: Delay in primordial germ cell migration in adamts9 knockout zebrafish
Source: Sci Rep. 2021 Apr 20;11:8545. doi: 10.1038/s41598-021-88024-x (PMC8058341; doi:10.1038/s41598-021-88024-x)
Supplement: Supplementary file 1 — Supplementary Information [file 41598_2021_88024_MOESM1_ESM.pdf]

Delay in Primordial Germ Cell Migration in Adamts9 Knockout Zebrafish

Jonathan J Carver , Yuanfa He, Yong Zhu

Supplemental Fig 1. Amino acid sequence alignments of human ADAMTS9 (hAdamts9, NP_891550), mice ADAMTS9 (mAdamts9, NP_780523), zebrafish Adamts9 (zAdamts9, NP_001244125), their ortholog in *Drosophila melanogaster* (dAdamTS-A, NP_996218) and *C. elegans* (cGON-1,NP_001255448). Metalloproteinase activation sites are highlighted in green color. Red arrow indicates the position of frame shift mutation caused by CRISPR/Cas9 genome editing that led to a premature stop codon in zebrafish Adamts9 (see ref #24 for detail).

```
dAdamTS-A      MSMPDAGSLKSPAGGQEVECHRLLSDLPAFAYPSTTL--RSTTSGH--STGTNSTTVCES 56
cGON-1         ------------------------------------------------------------ 0
zAdamts9       ----------------------MLSKLQEFGAYEIVTPARLNEVGEQLPTGVHFKR---- 34
hAdamts9       ------------------------------------------------------------ 0
mAdamts9       ------------------------------------------------------------ 0

dAdamTS-A      PGARDRTSFACSSSCVSSACSATASDDDEDERALERCLR--GTTDDLGESGLRYSLKGPP 114
cGON-1         ----------------------------------------------MRSIGGSFHLLQPV 14
zAdamts9       ---RKRS-------------------TDPTTANISHHWTSPHAYYQISAFGQDYYLNLTL 72
hAdamts9       ------------------------------------------------------------ 0
mAdamts9       ------------------------------------------------------------ 0

dAdamTS-A      PELYSAKKDFISKGPLLGRQLEVK--------KRCEWWCHKYI--QKMSTHWRQNACLYA 164
cGON-1         V-----------------------------------------------------------A 16
zAdamts9       ESGFIAP---VYTVTILGASSEGHNSVEGEEEEDTEYQHCFYKGHVNAGQEH-------T 122
hAdamts9       ------------------------------------MQF----------VSW-------A 7
mAdamts9       ------------------------------------MQL----------VSW-------A 7
                                                                          :
dAdamTS-A      CCIAFLLGMLIMFHLGLRSAHKGQEELPQSTHPLAN-------SPPATPATLHPRRLDND 217
cGON-1         ALILLVVCLVYAL----QSGSGTISEF--SSDVLF--SRAKYSGVP-VHHSRWRQDAGIH 67
zAdamts9       AVISLCSGLLGTF----RSPEGE---F--FVEPLHSYNSEHYEEEHIKPHVVYRKDASKK 173
hAdamts9       TLLTLLVRDLAEM----GSPD-----------------------------------AAAA 28
mAdamts9       TLLTLLVPDLVEM----RSPD-----------------------------------SAAA 28
                 : :    :  :     *

dAdamTS-A      TSTDHEPPDGLDDLDEEEHSAFVMPTKVYNYSLSE----ADLIYESKRNSDINSFLKESA 273
cGON-1         VIDSHHIVR-RDSYGRRGKRDVTSTDRRRRLQGVARDCGHACHLRLRSDDAV--Y----I 120
zAdamts9       TVDDSAACETSAKLQEFGAYEIVTP---ARLNEVGEQLPTGVHFKRRKRS----T----D 222
hAdamts9       VRKDRLHPRQVKLLETLSEYEIVSP---IRVNALGEPFPTNVHFKRTRRS----I----N 77
mAdamts9       VRKDRLHPRQVKLLESLSEYEIASP---IRVNPLGEPFPTNVHFKRRRRS----I----N 77
               .  .                 ..      . .            .    .

                                        prodomain
dAdamTS-A      SAFAMTGTYRNMSNEIWDPHPQYNLNVFGRQLHLVLRQDASFVHNHSMTHIRILK-EGEE 332
cGON-1         VHLHRWNQIPD---SHNKSVPHFSNSNFAPMVLY-------------------LD--SE- 155
zAdamts9       PTTA------NISHHWTSPHAYYQISAFGQDYYLNLTLESGFIAPVYT--VTILGASSEG 274
hAdamts9       SATDPWPAFASSSSSSTSSQAHYRLSAFGQQFLFNLTANAGFIAPLFT--VTLLGTPGVN 135
mAdamts9       SASDPWPAFASSSSSSTSSQEHYRLSAFGQQFLFNLTAHTGFIAPLFT--VTLLGEPGVN 135
                         .      .    :  . *.                        *   .

dAdamTS-A      HPGPETEAEAEQRHLGCFYSGYVEDDPHSMVSVSLC---GGMTGYIKTSFGALLIQPVNR 389
cGON-1         ---EEVRGGMSRTDPDCIYRAHVKGVHQHS-IVNLCDSEDGLYGMLALPSGIHTVEPIIS 211
zAdamts9       HNSVEGEEDEDTEYQHCFYKGHVNAGQEHTAVISLC---SGLLGTFRSPEGEFFVEPLHS 331
hAdamts9       QTK--FYSEEEAELKHCFYKGYVNTNSEHTAVISLC---SGMLGTFRSHDGDYFIEPLQS 190
mAdamts9       QTK--LYSEEEMELRHCFYKGHVNTKSEHTAVISLC---SGMMGTFRSHDGDYFIEPLQS 190
                         .     *:* .:*:   .    :.**   .*: * :    *   ::*:

dAdamTS-A      TSSDEV------LHRVFRKSQRN-----ARHAVSKFEL---------------------- 416
cGON-1         GNGTEHDGASRHRQHLVRKFDPMHFKS--------FDHLNSTSVNETETTV--------- 254
zAdamts9       YNSERYEEEHIKPHVVYRKDASKKTV-DDSAACETSGHTEPNRRHRNRL---KRKSPSSM 387
hAdamts9       MDEQEDEEEQNKPHIIYRRSAPQREPSTGRHACDTSEHKNRHSKDKKKTRARKWGERINL 250
mAdamts9       VDEQEDEEEQNKPHIIYRHSTPQREPSTGKHACATSELKNSHSKDKRKIRMRKRRKRNSL 250
                .  .        : : *:
```

Delay in Primordial Germ Cell Migration in Adamts9 Knockout Zebrafish

Jonathan J Carver , Yuanfa He, Yong Zhu

```
                                                               |-------------
dAdamTS-A       ---------GLDDFMSKLEQVQEEEQKSKSRKLNRKKRHYADVDNQVYTLEVLIAVDNSM 467
cGON-1          ------------------ATWQDQWEDVI-ERKARSRRA---ANSWDHYVEVLVVADTKM 292
zAdamts9        LSDLETLNSRLFPFS---ENKHNSANESSDSKPHRRSKR---FLSYPRFVEVMVVADSKM 441
hAdamts9        AGDVAALNSGLATEA--FSAYGNKTDNTREKRTHRRTKR---FLSYPRFVEVLVVADNRM 305
mAdamts9        ADDVALLKSGLATKV--LSGYSNQTNSTRDRWNHKRTKR---FLSYPRFVEVMVVADHRM 305
                                      :. :.       :  :      .    :**::..*  *

                ------------------------------------------------------------
dAdamTS-A       KQFHGEDLQPYILILMSIVSSIFADASIGNSIRILLVRLISLPNINDQT---HSSNEMLK 524
cGON-1          YEYHGRSLEDYVLTLFSTVASIYRHQSLRASINVVVVKLIVLKTENAGPRITQNAQQTLQ 352
zAdamts9        VEHHGSNLQHYILTLMSIVSSIYKDPSIGNLINIVIVKLVIIKNELDGPTISFNAQATLK 501
hAdamts9        VSYHGENLQHYILTLMSIVASIYKDPSIGNLINIVIVNLIVIHNEQDGPSISFNAQTTLK 365
mAdamts9        VLYHGANLQHYILTLMSIVASIYKDSSIGNLINIVIVNLVVIHNEQEGPYINFNAQTTLK 365
                  .** .*: *:* *:* *:**: . *:   *.:::*.*: : .         .::  *:

                ------------------ metalloprotease domain -----------↓------
dAdamTS-A       HFCQFINQSG-------YERDTAMLITREPICGSVPGKICHMLGLAELGTVCSS-SSCSI 576
cGON-1          DFCRWQQYYNDPDDSSVQHHDVAILLTRKDICRSQ--GKCDTLGLAELGTMCDMQKSCAI 410
zAdamts9        NFCIWQQSQNHPDDNHPSHHDTAILITRQDICRAR--DKCDTLGLAELGTVCDPYRSCSI 559
hAdamts9        NFCQWQHSKNSPGG---IHHDTAVLLTRQDICRAH--DKCDTLGLAELGTICDPYRSCSI 420
mAdamts9        NFCQWQHSKNYLGG---IQHDTAVLVTREDICRAQ--DKCDTLGLAELGTICDPYRSCSI 420
                .** : :  .        .:*.*:*:**: ** :     *. ********:*.   **:*

                ------------------------------------------------------------
dAdamTS-A       VQDTGLPTAFTMAHELGHILNMNHDDDDKCMPYVTRQNNNKVLHIMSSVMGIHMHPWSWS 636
cGON-1          IEDNGLSAAFTIAHELGHVFSIPHDDERKCSTYMPVNK--NNFHIMAPTLEYNTHPWSWS 468
zAdamts9        NEDNGLSTAFTIAHELGHVFNMPHDDSNKCKEDG--VK--NQQHVMAPTLNYYTNPWMWS 615
hAdamts9        SEDSGLSTAFTIAHELGHVFNMPHDDNNKCKEEG--VK--SPQHVMAPTLNFYTNPWMWS 476
mAdamts9        SEDSGLSTAFTIAHELGHVFNMPHDDSNKCKEEG--VK--SPQHVMAPTLNFYTNPWMWS 476
                 :*.** :***:******::.: ***. **       :  .  *:*: .:    :** **

                -------------------------|
dAdamTS-A       KCSRHFVSEFLEKT--DKSCLETSVGAHIPY---GTERLPGEIYSLDAQCQLSFGNDFGY 691
cGON-1          PCSAGMLERFLENNRGQTQCLFDQPVERRYYEDVFVRDEPGKKYDAHQQCKFVFGPASEL 528
zAdamts9        KCSRKYITEFLDTG--YGECLLDEPVSRPYSL---SQQLPGQIYSVNKQCELIFGPGTQV 670
hAdamts9        KCSRKYITEFLDTG--YGECLLNEPESRPYPL---PVQLPGILYNVNKQCELIFGPGSQV 531
mAdamts9        KCSRKYITEFLDTG--YGECLLNEPASRTYPL---PSQLPGLLYNVNKQCELIFGPGSQV 531
                 **   : .**:.     .**  .   :           **  *. . **:: **

dAdamTS-A       CPTDEECKRLWCNRTSGNSNEQCASSNLPWADGTPCGSSG-HWCQRGKCVSNKHGYGRQV 750
cGON-1          CPYMPTCRRLWCATFYGS-QMGCRTQHMPWADGTPCDESRSMFCHHGACVRLAPESLTKI 587
zAdamts9        CPYMTQCRRLWCTSPDGV-QRGCRTQHMPWADGTDCAPGK--HCKHGLCIHKEHE-YVPV 726
hAdamts9        CPYMMQCRRLWCNNVNGV-HKGCRTQHTPWADGTECEPGK--HCKYGFCVPKEMD-VPVT 587
mAdamts9        CPYMMQCRRLWCNNVDGA-HKGCRTQHTPWADGTECEPGK--HCKFGFCVPKEME-GPAI 587
                **    *:****    *  :  * :.: ****** *  .    *: * *:

                   |------- thrombospondin type 1 repeats (TSP-1) ---------
dAdamTS-A       NGGWGPWTPFTPCSLTCGGGVQESRRECNQP---------------VPENGGKYCTGSRK 795
cGON-1          DGQWGDWRSWGECSRTCGGGVQKGLRDCDSPKSENEVETLKSNVTSRPRNGGKYCVGQRE 647
zAdamts9        EGAWGVWSPFGTCSRTCGGGIKIAVRECNRP---------------VPRNGGKYCVGRRM 771
hAdamts9        DGSWGSWSPFGTCSRTCGGGIKTAIRECNRP---------------EPKNGGKYCVGRRM 632
mAdamts9        DGSWGGWSHFGTCSRTCGGGIKTAIRECNRP---------------EPKNGGKYCVGRRM 632
                :* ** *  :  ** *****:: . *:*: *                *.******.* *

                -----------|
dAdamTS-A       KYRSCNTHQCPPGSMDPREQQCYAMNGRNMNIPGVN-PDTKWVPKYE----KDACKLFCR 850
cGON-1          RYRSCNTQECPWDTQPYREVQCSEFNNKDIGIQGVASTNTHWVPKYANVAPNERCKLYCR 707
zAdamts9        KFRSCNSEPCSKQKKDFREEQCASFDGRHFNINGLP-PNVRWVPKYSGILMKDRCKLFCR 830
hAdamts9        KFKSCNTEPCLKQKRDFRDEQCAHFDGKHFNINGLL-PNVRWVPKYSGILMKDRCKLFCR 691
mAdamts9        KFKSCNTEPCMKQKRDFREEQCAHFDGKHFNINGLL-PSVRWVPKYSGILMKDRCKLFCR 691
                :::***:. *   .   *: **  ::.:.:.* *:   ..:*****     :: ***:**
```

Delay in Primordial Germ Cell Migration in Adamts9 Knockout Zebrafish

Jonathan J Carver , Yuanfa He, Yong Zhu

```
dAdamTS-A     MDMKVTYFMLKSMVTDGTSCAVDSFDKCVNGICRPAGCDNELNSIAKLDKCGVCEGRNDT  910
cGON-1        LSGSAAFYLLRDKVVDGTPCDRNGDDICVAGACMPAGCDHQLHSTLRRDKCGVCGGDDSS  767
zAdamts9      VAGSTAYYQLRDRVTDGTQCGPDTNDICVQGLCRQAGCDHVLNSKARRDKCGVCGGDNSS  890
hAdamts9      VAGNTAYYQLRDRVIDGTPCGQDTNDICVQGLCRQAGCDHVLNSKARRDKCGVCGGDNSS  751
mAdamts9      VAGNTAYYQLRDRVIDGTPCGQDTNDICVQGLCRQAGCDHILNSKARKDKCGICGGDNSS  751
              :  ..::: *:. * *** *  :  * ** * *  ****: *:*  : ****:* * :.:

dAdamTS-A     CHEVTGNLLVSNLLGLNDGNEPNKTLYYVTRIPKGASNIIITQRGYP----DQNFIVLTD  966
cGON-1        CKVVKGTFNEQGTFGYN----------EVMKIPAGSANIDIRQKGYNNMKEDDNYLSLRA  817
zAdamts9      CKPVAGTFNIV-HYGYN----------VVVRIPSGATNIDVRQHSYSGKAEDDNYLALSN  939
hAdamts9      CKTVAGTFNTV-HYGYN----------TVVRIPAGATNIDVRQHSFSGETDDDNYLALSS  800
mAdamts9      CKTVAGTFNTV-HYGYN----------TVVRIPAGATSIDVRQHSFSGKSEDDNYLALSN  800
              *: * *.:      * *           * :** *::.* : *:.:     *:*:: *

                                   spacer
dAdamTS-A     DRDNELLNGKFLKTYPLK-FVYAGVTMQYTGSSSVVEQVNTTYSWKLSRDLIVQIISLDV  1025
cGON-1        ANGEFLLNGHFQVSLARQQIAFQDTVLEYSGSDAIIERINGTGP--IRSDIYVHVLSVGS  875
zAdamts9      SRGEYLLNGDFVVSMFKREVRVGNAVIEYSGSDHVVERINCTDR--IEEEIIIQVLSVGN  997
hAdamts9      SKGEFLLNGNFVVTMAKREIRIGNAVVEYSGSETAVERINSTDR--IEQELLLQVLSVGK  858
mAdamts9      SKGEFLLNGDFVVSMSKREVRVGSAVIEYSGSDNVVERLNCTDR--IEEELLLQVLSVGK  858
               ..: ****.*  :   : .   ...::*:**.  :*::* *    :  :: ::::*:.

dAdamTS-A     SPSKRQDTVLLSYSYTIDKPPDYE---AEVEIYRWEMQAP-SNCDSLCEGRSHRLPACIS  1081
cGON-1        H------PPDISYEYMTAAVPNAVIRPISSALYLWRVTDTWTECDRACRGQQSQKLMCLD  929
zAdamts9      L-----YNPDVRYSYNIPIE-------DKPQHFFWDAYGPWQDCSLLCQGERKKKILCNR  1045
hAdamts9      L-----YNPDVRYSFNIPIE-------DKPQQFYWNSHGPWQACSKPCQGERKRKLVCTR  906
mAdamts9      L-----YNPDVRYSFNIPIE-------DKPQQFYWNSHGPWQACSKPCQGERRRKLVCTR  906
                        : *.:              .   : *        *.  *.*.  :   *

dAdamTS-A     TTQGVKVAPQFCDKSAMPKIDDRACNTDCRLNLTVTSISECSAACGELGTREKTYACVQT  1141
cGON-1        MSTHRQSHDRNCQNVLKPKQATRMCNIDCSTRWITEDVSSCSAKCGSGQ-KRQRVSCVKM  988
zAdamts9      ESDRVVVSDQRCHGLPKPAAITESCNTDCELGWHIARKSECTAACGVGY-RSLDIYCTKQ  1104
hAdamts9      ESDQLTVSDQRCDRLPQPGHITEPCGTDCDLRWHVASRSECSAQCGLGY-RTLDIYCAKY  965
mAdamts9      ESDQLTVSDQRCDRLPQPGPVTEACGTDCDLRWHVASKSECSAQCGLGY-RTLDIHCAKY  965
               :       : *.    *    . *. **         *.*:* **    :     *.:

dAdamTS-A     FTNMQRSNIVDMSYCKLKFDVAYHE----ECR---------------------------  1169
cGON-1        EGD--RQTPASEHLCDRNSKPSDIASCYIDCSGRKWNYGEWTSCSETCGSNGKMHRKSYC  1046
zAdamts9      SRLDGKTQKVDERYCSSQHKPNDKEVCHGDCNP---------------------------  1137
hAdamts9      SRLDGKTEKVDDGFCSSHPKPSNREKCSGECNT---------------------------  998
mAdamts9      SRMDGKTEKVDDSFCSSQPRPSNQEKCSGECST---------------------------  998
                   :   ..   *. :           :*

                      TSP-1
dAdamTS-A     ------------------------------------------------------------
cGON-1        VDDSNRRVDESLCGREQKEATERECNRIPCPRWVYGHWSECSRSCDGGVKMRHAQCLDAA  1106
zAdamts9      ------------------------------------------------------------
hAdamts9      ------------------------------------------------------------
mAdamts9      ------------------------------------------------------------

                      TSP-1
dAdamTS-A     ------------------------------------------------------------  1197
cGON-1        DRETHTSRCGPAQTQEHCNEHACTWWQFGVWSDCSAKCGDGVQYRDANCTDRHRSVLPEH  1166
zAdamts9      -----------------------------------------------------         1173
hAdamts9      -----------------------------------------------------         1035
mAdamts9      -----------------------------------------------------         1035
```

```
                      TSP-1         |                    TSP-1
dAdamTS-A      ------------------------------------------------------------ 1197
cGON-1         RCLKMEKIITKPCHRESCPKYKLGEWSQCSVSCEDGWSSRRVSCVSGNGTEVDMSLCGTA 1226
zAdamts9       --------------------GGWEYSSWSECSRSCGGGTRRRNAICGKS-DERDDDSKCN 1176
hAdamts9       --------------------GGWRYSAWTECSKSCDGGTQRRRAICVNTRNDVLDDSKCT 1038
mAdamts9       --------------------GGWRYSAWTECSRSCDGGTQRRRAICVNTRNDVLDDSKCT 1038

                                |    |                TSP-1
dAdamTS-A      --------------EGCWVLSEWSTCSKSCGTGSQQREAHCYLHNSRVSDDLCNPRTKPH 1215
cGON-1         SDRPASHQTCNLGTCPFWRNTDWSACSVSCGIGHRERTTECIYREQSVDASFCGDTKMPE 1286
zAdamts9       PQEKLTAQPCNEFLCPQWKTGDWSECLVTCGKGYKHRQTWCQFGEERLDVRFCDS-SKPE 1235
hAdamts9       HQEKVTIQRCSEFPCPQWKSGDWSECLVTCGKGHKHRQVWCQFGEDRLNDRMCDPETKPT 1098
mAdamts9       HQEKVVVQSCNEFSCPHWKTGDWSECLVTCGKGHKHRQVWCQFGEDRLSDRMCDPEAKPE 1098
                                *   :**.* .:**.*       *   :. :.  :*.    *

                     |         |                  TSP-1
dAdamTS-A      LNTLI-------------------------------------------------------G 1221
cGON-1         TSQTCHLLPCTSWKPSHWSPCSVTCGSGIQTRSVSCTRGSEGTIVDEYFCDRNTRPRLKK 1346
zAdamts9       SVQACQQQECASWQVGPWGQCTTTCGPGYQMRAVKCVVGSYGSVMDDTECNAATRPTDTQ 1295
hAdamts9       SMQTCQQPECASWQAGPWGQCSVTCGQGYQLRAVKCIIGTYMSVVDDNDCNAATRPTDTQ 1158
mAdamts9       PMQTCQQPECAAWQAGPWGQCSVTCGQGYQLRAVKCIMGTYMSVVDDNDCNAATRPTDTQ 1158

                      |                    |                 TSP-1
dAdamTS-A      ICNTESCP-------TYTKSPNALAVSNWVIGEWGECNEWCEK----------------- 1257
cGON-1         TCEKDTCDGPRVLQ----KLQADVPPIRWATGPWTACSATCGNGTQRRLLKCRDHVRDLP 1402
zAdamts9       DCGLSQCPVTHPVAPEPKVMPHPGHKTQWRFGSWTQCSATCGKGTRMRYVSCRDQQGGVA 1355
hAdamts9       DCELPSCH-PPPAAPETRRSTYSAPRTQWRFGSWTPCSATCGKGTRMRYVSCRDENGSVA 1217
mAdamts9       DCELASCH-PSILALEPRRNAQSIPRTQWRFGSWTPCSATCGKGTRMRYVSCRDEDGSVA 1217
                *    *                    .*  * *  *.  * :

                                     |  |                TSP-1
dAdamTS-A      ------------------------------------------------------------ 1257
cGON-1         DE-YCNHLDKEVSTRNCRLRDCSYWKMAEWEECPATCGTHVQQSRNVTCVSAEDGGRTIL 1461
zAdamts9       EESACAHLPKPPASEVCSIVACGQWKVLEWTACSVSCGQGTT-TRQVVCMNISD---QVV 1411
hAdamts9       DESACATLPRPVAKEECSVTPCGQWKALDWSSCSVTCGQGRA-TRQVMCVNYSD---HVI 1273
mAdamts9       DESACATLPKPVAKEECSVTPCGQWKALDWSSCSVTCGQGKA-TRQVVCVNYSD---HVI 1273

                                       |
dAdamTS-A      ------------------------TRSVSCSHPYGIGCGSRKPKDVRKCCH -------- 1284
cGON-1         KDVDCDVQKRPTSARNCRLEPCPKGEE--------------------------------H 1489
zAdamts9       ELSECDLDDKPAAEQECAMPQCPSRSSDHGGFS---PNPDFRKKTALPGRTDRNRAGRLQ 1468
hAdamts9       DRSECDQDYIPETDQDCSMSPCPQRTPDSGLAQHPFQNEDYRPRSAS-----PSRTHVLG 1328
mAdamts9       DRSECDPDYIPETDQDCSMSPCPQWTG----LAHPFQNEDFRPRSDS-----PSRTHVLG 1324

                  |                          TSP-1                    |
dAdamTS-A      ------------------------------------------------------------ 1284
cGON-1         IGSWIIGDWSKCSASCGGGWRRRSVSCT------SSSCDETRKPKMFDKCNEELCPPLTN 1543
zAdamts9       AQQWRTGPWGACSSTCAGGFQRRVVVCQDENGYPASSCDESIQPIEQRSCESGSCP---- 1524
hAdamts9       GNQWRTGPWGACSSTCAGGSQRRVVVCQDENGYTANDCVERIKPDEQRACESGPCP---- 1384
mAdamts9       GNQWRTGPWGACSSTCAGGSQRRVVVCQDENGYTANDCVERIKPDEQRACESGPCP---- 1380

                 |                            TSP-1
dAdamTS-A      -IKYTSDWTDCSVQCGEGVKRKKQSCTRVYKPDVPGTRKRRVY---VDESYCISR----- 1335
cGON-1         NSWQISPWTHCSVSCGGGVQRRKIWCEDVLSGRKQDDIECSEIK-PREQRDCEMPPCRSH 1602
zAdamts9       -QWFYGSWSECSKSCGGGIKTRLVACQRPN-GERFNDLSCEILDKPPDREQCNTQ----- 1577
hAdamts9       -QWAYGNWGECTKLCGGGIRTRLVVCQRSN-GERFPDLSCEILDKPPDREQCNTH----- 1437
mAdamts9       -QWAYGSWGECTKLCGGGMRTRLVVCQRAN-GDRFPDLSCEVLDKPTDREQCNTH----- 1433
                    . * .*:  ** *:: :   *            .        :.  *

                                                   |   |
dAdamTS-A      K-----------------VHRPKLRTTTKSCRINCKWNASDWRRCPADC----------- 1367
cGON-1         YHNKTSSASMTSLSSSNSNTTSSASASSLPILPPVVSWQTSAWSACSAKCGRGTKRRVVE 1662
zAdamts9       ------------------------------SCSINPHWSTDQWSLCFASCWSLHSSHLDQ 1607
```

Delay in Primordial Germ Cell Migration in Adamts9 Knockout Zebrafish

Jonathan J Carver , Yuanfa He, Yong Zhu

```
hAdamts9     ------------------------------ACPHDAAWSTGPWSSCSVSCGRGHKQRNVY 1467
mAdamts9     ------------------------------ACPQDAAWSTGPWSSCSVSCGRGHKHRNVY 1463
                                               *.:. *  * ..*

                       TSP-1
dAdamTS-A    ------------------------------------------------------SEEYQT 1373
cGON-1       CVNPS-------LNVTVASTECDQTKKPVEEVRCRTKHCPRWKTTTWSSCSVTCGRGIRR 1715
zAdamts9     VGLQISSGESGKGGDEVDRGFFKNKD------MGGGPKETDW------------------ 1643
hAdamts9     C--------MAKDGSHLESDYCKHLAKPHGHRKCRGGRCPKWKAGAWSQCSVSCGRGVQQ 1519
mAdamts9     C--------LAKDGSHLESDNCKHLPKPHGHRRCRGGRCPRWKAGAWSQCSVSCGQGVQQ 1515

                           TSP-1
dAdamTS-A    RDVRC------------------------------------------------------- 1378
cGON-1       REVQCYRGRKNLVSDSECNPKTKLNSVANCFPVACPAYRWNVTPWSKCKDECARGQKQTR 1775
zAdamts9     ------------------------------------------------------------ 1643
hAdamts9     RHVGCQIGTHKIARETECNPYTRPESERDCQGPRCPLYTWRAEEWQECTKTCGEGSRY-R 1578
mAdamts9     RHVGCQIGTHKAARESECSSYSRPESERVCQASPCPLYTWRAEQWQQCTKTCGEGSRY-R 1574

                             TSP-1
dAdamTS-A    ----ESFQGDGVEDKHCDAKKRPSKRRICNNCVRRQSRVI------SQCNCEGVEKRRDF 1428
cGON-1       RVHCISTSGKRAAPRMCELARAPTSIRECDTSNCPYEWVPGDWQTCSKSCGEGVQTREVR 1835
zAdamts9     ------------------------------------------------------------ 1643
hAdamts9     KVVCVDDNKNEVHGARCDVSKRPVDRESCSLQPCEYVWITGEWSECSVTCGKGYKQRLVS 1638
mAdamts9     QVVCVAEDQSEVHSTHCDSDQRPPDRESCSLQPCEYVWITGEWSECSVTCGKGYRQRLVS 1634

                               TSP-1
dAdamTs-A    CFNSHKGRIACPTRARVERHSCTPPPHCRR------------RS-------AIGS-SISS 1468
cGON-1       CRRKINFNSTIPIIFMLEDEPAVPKEKCELFPKPNESQTCELNPCDSEFKWSFGPWGECS 1895
zAdamts9     ------------------------------------------------------------ 1643
hAdamts9     CSEIYTGKENYEYSYQ--TTI-----NCP-GTQPPSVHPCYLRDCPVSATWRVGNWGSCS 1690
mAdamts9     CSEIYTGKENYEYSYQ--TTV-----NCP-GAQPPSVHPCYLRDCPVSATWRVGNWGSCS 1686

                            TSP-1
dAdamTS-A    RPRGTGVSS----------------------SRSLNSIGGSRNRGTPRSCADLKEMHGYN 1506
cGON-1       KNCGQGIRRRRVKCVANDGRRVERVKCTTKKPRRTQYC-FERNC-LPSTCQELKSQNVKA 1953
zAdamts9     ------------------------------------------------------------ 1643
hAdamts9     VSCGVGVMQRSVQCLTNEDQPSHLCHTDL-KPEERKTCRNVYNCELPQNCKEVKRLKGAS 1749
mAdamts9     VSCGIGVMHRSVQCLTNEDQPSHLCPTDT-KPEERKACRNVYNCELPQNCKEVKKLNSAS 1745

dAdamTs-A    KDGNYQLEVRSRMVHIYCHGMNSRTPQEYVNVD--PQENYSIYYEYRTKQTNSCPPESRG 1564
cGON-1       KDGNYTILLDGFTIEIYCHRMNSTIPKAYLNVN--PRTNFAEVYGKKLIYPHTCPFNGDR 2011
zAdamts9     ------------------------------------------------------------ 1643
hAdamts9     EDGEYFLMIRGKLLKIFCAGMHSDHPKEYVTLVHGDSENFSEVYGHRLHNPTECPYNGSR 1809
mAdamts9     VDGEYFLAVRGKPLKVFCAGMNSDYPKEYVTLAHGDSENFSEVYGHRLHNPTECPYNGSR 1805

                                     gon-1 domain
dAdamTS-A    HEY-----YNDQNSGRTHFRKLRLNITDLRIMDNDFKFAD-SRGLAQKLGSAGDCYNRIG 1618
cGON-1       NDSCHCSEDGDASAGLTRFNKVRIDLLNRKFHLADYTFAKREYGVHVPYGTAGDCYS-MK 2070
zAdamts9     ------------------------------------------------------------ 1643
hAdamts9     RDDCQCRKDY-TAAGFSSFQKIRIDLTSMQIITTDLQFARTSEGHPVPFATAGDCYS-AA 1867
mAdamts9     RDDCHCRKDY-TAAGFSSFQKIRLDLTSMQIITTDLEFARTSEGHPVPFATAGDCYS-AA 1863

dAdamTS-A    QCPQGDFSINMKDTDFSIRPGTVWRMHGQYSVMKRISEFDTTTQMRRGFCGGYCGGCYIA 1678
cGON-1       DCPQGIFSIDLKSAGLKLVDDLNWEDQGHRTSSRIDRFYNNA--KVIGHCGGFCGKCSPE 2128
zAdamts9     ------------------------------------------------------------ 1643
hAdamts9     KCPQGRFSINLYGTGLSLTESARWISQGNYAVSDIKKSPDGT--RVVGKCGGYCGKCTPS 1925
mAdamts9     KCPQGRFSINLYGTGLSLTESARWTSQGNYAVSDIKKSPDGT--RVVGKCGGYCGKCTPS 1921

dAdamTS-A    PDSGLYLDVL--------------------------        1688
```

Delay in Primordial Germ Cell Migration in Adamts9 Knockout Zebrafish
Jonathan J Carver , Yuanfa He, Yong Zhu

```
cGON-1        RYKGLIFEVNTKLLNHVKNGGHIDDELDDDGFSGDMD        2165
zAdamts9      -------------------------------------        1643
hAdamts9      SGTGLEVRVL---------------------------        1935
mAdamts9      SGTGLEVRVS---------------------------        1931
```

Supplemental Figure 2. Amino acid sequences and functional domains (highlighted in different color) of Adamts9 in human, mouse, zebrafish, and their ortholog in *Drosophila* and *C. elegans*

Signal peptide, highlighted in red color;
Prodomain, highlighted dark blue color;
Metalloproteinase domain, highlighted in grey color;
Thrombospondin type 1 repeats (TSP-1), highlighted in yellow color;
Spacer, highlighted in light blue color;
GON-1 domain, highlighted in purple color;

>hAdamts9 NP_891550.1 *Homo sapiens* ADAMTS9 amino acid sequence
MQFVSWATLLTLLVRDLA
EMGSPDAAAAVRKDRLHPRQVKLLETLSEYEIVSPIRVNALGEPFPTNVHFKRT
RRSINSATDPWPAFASSSSSSTSSQAHYRLSAFGQQFLFNLTANAGFIAPLFTVTLLGTPGVNQTKFYSEEEAELKH
CFYKGYVNTNSEHTAVISLCSGMLGTFRSHDGDYFIEPLQSMDEQEDEEEQNKPHIIY
RRSAPQREPSTGRHACDTSEHKNRHSKDKKKTRARKWGERINLAGDVAALNSGLATEAFSAYGNKTDNTREKRTHRR
TKRFLSYP
RFVEVLVVADNRMVSYHGENLQHYILTLMSIVASIYKDPSIGNLINIVIVNLIVIHNEQDGPSISFNAQTTLKNFCQ
WQHSKNSPGGIHHDTAVLLTRQDICRAHDKCDTLGLAELGTICDPYRSCSISEDSGLSTAFTIAHELGHVFNMPHDD
NNKCKEEGVKSPQHVMAPTLNFYTNPWMWSKCSRKYITEFLDTGYGECLLNEP
ESRPYPLPVQLPGILYNVNKQCELIFGPGSQVCPYMMQCRRLWCNNVNGVHKGCRTQHTPWADGTECEPGKHCKYGF
CVPKEMDVPVTDGS
WGSWSPFGTCSRTCGGGIKTAIRECNRPEPKNGGKYCVGRRMKFKSCNTEPC
LKQKRDFRDEQCAHFDGKHFNINGLLPNVRWVPKYSGILMKDRCKLFCRVAGNTAYYQLRDRVIDGTPCGQDTNDIC
VQGLCRQAGCDHVLNSKARRDKCGVCGGDNSSC
KTVAGTFNTVHYGYNTVVRIPAGATNIDVRQHSFSGETDDDNYLALSSSKGEFLLNGNFVVTMAKREIRIGNAVVEY
SGSETAVERINSTDRIEQELLLQVLSVGKLYNPDVRYSFNIP
IEDKPQQFYWNSHGPWQACSKPCQGERKRKLVCTRESDQLTVSDQRCDRLPQPGHITEPCGTDCDLRWHVASRSECS
AQCGLGYRTLDIYCAKYSRLDGKTEKVDDGFCSSHPKPSNREKCSGECNTGG
WRYSAWTECSKSCDGGTQRRRAICVNTRNDVLDDSKCTHQEKVTIQRCSEFPCP
QWKSG
DWSECLVTCGKGHKHRQVWCQFGEDRLNDRMCDPETKPTSMQTC
QQPECASWQA
GPWGQCSVTCGQGYQLRAVKCIIGTYMSVVDDNDCNAATRPTDTQDCELPSCH
PPPAAPETRRSTYSAPRTQ
WRFGSWTPCSATCGKGTRMRYVSCRDENGSVADESACATLPRPVAKEECSVTP
CGQ
WKALDWSSCSVTCGQGRATRQVMCVNYSDHVIDRSECDQDYIPETDQDCSMSPCP
QRTPDSGLAQHPFQNEDYRPRSASPSRTHVLGGNQ
WRTGPWGACSSTCAGGSQRRVVVCQDENGYTANDCVERIKPDEQRACESGPCP
Q
WAYGNWGECTKLCGGGIRTRLVVCQRSNGERFPDLSCEILDKPPDREQCNTHACP
HDAA
WSTGPWSSCSVSCGRGHKQRNVYCMAKDGSHLESDYCKHLAKPHGHRKCRGGRCP
KWKA
GAWSQCSVSCGRGVQQRHVGCQIGTHKIARETECNPYTRPESERDCQGPRCP
LYTWRA
EEWQECTKTCGEGSRYRKVVCVDDNKNEVHGARCDVSKRPVDRESCSLQPCE
Y
VWITGEWSECSVTCGKGYKQRLVSCSEIYTGKENYEYSYQTTINCPGTQPPSVHPCYLRDCP
VSATWRVGNWGSCSVSCGVGVMQRSVQCLTNEDQPSHLCHTDLKPEERKTCRNVYNCELPQ
NCKEVKRLKGASEDGEYFLMIRGKLLKIFCAGMHSDHPKEYVTLVHGDSENFSEVYGHRLHNPTECPYNGSRRDDCQ
CRKDYTAAGFSSFQKIRIDLTSMQIITTDLQFARTSEGHPVPFATAGDCYSAAKCPQGRFSINLYGTGLSLTESARW
ISQGNYAVSDIKKSPDGTRVVGKCGGYCGKCTPSSGTGLEVRV

L
>mAdamts9 NP_780523.3 *Mus musculus* ADAMTS9 amino acid sequence
MQLVSWATLLTLLVPDLVEMRSPDSAAAVRKDRLHPRQVKLLESLSEY
EIASPIRVNPLGEPFPTNVHFKRRRRSINSASDPWPAFASSSSSSTSSQEHYRLSAFGQQFLFNLTAHTGFIAPLFT
VTLLGEPGVNQTKLYSEEEMELRHCFYKGHVNTKSEHTAVISLCSGMMGTFRSHDGDYFIEPLQSVDEQEDEEEQNK
PHIIY
RHSTPQREPSTGKHACATSELKNSHSKDKRKIRMRKRRKRNSLADDVALLKSGLATKVLSGYSNQTNSTRDRWNHKR
TK
RFLSYPRFVEVMVVADHRMVLYHGANLQHYILTLMSIVASIYKDSSIGNLINIVIVNLVVIHNEQEGPYINFNAQTT
LKNFCQWQHSKNYLGGIQHDTAVLVTREDICRAQDKCDTLGLAELGTICDPYRSCSISEDSGLSTAFTIAHELGHVF
NMPHDDSNKCKEEGVKSPQHVMAPTLNFYTNPWMWSKCSRKYITEFLDTGYGECLLNEP
ASRTYPLPSQLPGLLYNVNKQCELIFGPGSQVCPYMMQCRRLWCNNVDGAHKGCRTQHTPWADGTECEPGKHCKFGF
CVPKEMEGPAIDGS
WGGWSHFGTCSRTCGGGIKTAIRECNRPEPKNGGKYCVGRRMKFKSCNTEPC
MKQKRDFREEQCAHFDGKHFNINGLLPSVRWVPKYSGILMKDRCKLFCRVAGNTAYYQLRDRVIDGTPCGQDTNDIC
VQGLCRQAGCDHILNSKARKDKC
GICGGDNSSCKTVAGTFNTVHYGYNTVVRIPAGATSIDVRQHSFSGKSEDDNYLALSNSKGEFLLNGDFVVSMSKRE
VRVGSAVIEYSGSDNVVERLNCTDRIEEELLLQVLSVGKLYNPDVRYSFNIP
IEDKPQQFYWNSHGPWQACSKPCQGERRRKLVCTRESDQLTVSDQRCDRLPQPGPVTEACGTDCDLRWHVASKSECS
AQCGLGYRTLDIHCAKYSRMDGKTEKVDDSFCSSQPRPSNQEKCSGECSTGGW
RYSAWTECSRSCDGGTQRRRAICVNTRNDVLDDSKCTHQEKVVVQSCNEFSCP
HWKTGDWSECLVTCGKGHKHRQVWCQFGEDRLSDRMCDPEAKPEPMQTCQQPE
CAAWQA
GPWGQCSVTCGQGYQLRAVKCIMGTYMSVVDDNDCNAATRPTDTQDCELASC
HPSILALEPRRNAQSIPRTQ
WRFGSWTPCSATCGKGTRMRYVSCRDEDGSVADESACATLPKPVAKEECSVTP
CGQ
WKALDWSSCSVTCGQGKATRQVVCVNYSDHVIDRSECDPDYIPETDQDCSMSPCP
QWTGLAHPFQNEDFRPRSDSPSRTHVLGGNQ
WRTGPWGACSSTCAGGSQRRVVVCQDENGYTANDCVERIKPDEQRACESGPCP
Q
WAYGSWGECTKLCGGGMRTRLVVCQRANGDRFPDLSCEVLDKPTDREQCNTHACP
QDAA
WSTGPWSSCSVSCGRGHKHRNVYCLAKDGSHLESDNCKHLPKP
HGHRRCRGGRCPRWKA
GAWSQCSVSCGQGVQQRHVGCQIGTHKAARESECSSYSRPESERVCQASPCP
LYTWRAEQ
WQQCTKTCGEGSRYRQVVCVAEDQSEVHSTHCDSDQRPPDRESCSLQPCE
Y
VWITGEWSECSVTCGKGYRQRLVSCSEIYTGKENYEYSYQTTVNCPGAQPPSVHPCYLRDCP
VSATWRVGNWGSCSVSCGIGVMHRSVQCLTNEDQPSHLCPTDTKPEERKACRNVYNCELPQ
NCKEVKKLNSASVDGEYFLAVRGKPLKVFCAGMNSDYPKEYVTLAHGDSENFSEVYGHRLHNPTECPYNGSRRDDCH
CRKDYTAAGFSSFQKIRLDLTSMQIITTDLEFARTSEGHPVPFATAGDCYSAAKCPQGRFSINLYGTGLSLTESARW
TSQGNYAVSDIKKSPDGTRVVGKCGGYCGKCTPSSGTGLEVRV
S
>zAdamts9 NP_001244125.1 *Danio rerio* Adamts9 amino acid sequence
MLSKLQEFGAYEIVTPARLNEVGEQLPTGVHFKR
RKRSTDPTTANISHHWTSPHAYYQISAFGQDYYLNLTLESGFIAPVYTVTILGASSEGHNSVEGEEEEDTEYQHCFY
KGHVNAGQEHTAVISLCSGLLGTFRSPEGEFFVEPLHSYNSEHYEEEHIKPHVV
YRKDASKKTVDDSAACETSAKLQEFGAYEIVTPARLNEVGEQLPTGVHFKR
RKRSTDPTTANISHHWTSPHAYYQISAFGQDYYLNLTLESGFIAPVYTVTILGASSEGHNSVEGEEDEDTEYQHCFY
KGHVNAGQEHTAVISLCSGLLGTFRSPEGEFFVEPLHSYNSERYEEEHIKPHVV
YRKDASKKTVDDSAACETSGHTEPNRRHRNRLKRKSPSSMLSDLETLNSRLFPFSENKHNSANESSDSKPHRRSK
RFLSYP
RFVEVMVVADSKMVEHHGSNLQHYILTLMSIVSSIYKDPSIGNLINIVIVKLVIIKNELDGPTISFNAQATLKNFCI
WQQSQNHPDDNHPSHHDTAILITRQDICRARDKCDTLGLAELGTVCDPYRSCSINEDNGLSTAFTIAHELGHVFNMP
HDDSNKCKEDGVKNQQHVMAPTLNYYTNPWMWSKCSRKYITEFLDTGYGECLLDEP

VSRPYSLSQQLPGQIYSVNKQCELIFGPGTQVCPYMTQCRRLWCTSPDGVQRGCRTQHMPWADGTDCAPGKHCKHGL
CIHKEHEYVPVEGA
WGVWSPFGTCSRTCGGGIKIAVRECNRPVPRNGGKYCVGRRMKFRSCNSEPCS
KQKKDFREEQCASFDGRHFNINGLPPNVRWVPKYSGILMKDRCKLFCRVAGSTAYYQLRDRVTDGTQCGPDTNDICV
QGLCRQAGCDHVLNSKARRDKCGVCGGDNSSC
KPVAGTFNIVHYGYNVVVRIPSGATNIDVRQHSYSGKAEDDNYLALSNSRGEYLLNGDFVVSMFKREVRVGNAVIEY
SGSDHVVERINCTDRIEEEIIIQVLSVGNLYNPDVRYSYNIP
IEDKPQHFFWDAYGPWQDCSLLCQGERKKKILCNRESDRVVVSDQRCHGLPKPAAITESCNTDCELGWHIARKSECT
AACGVGYRSLDIYCTKQSRLDGKTQKVDERYCSSQHKPNDKEVCHGDCNPGG
WEYSSWSECSRSCGGGTRRRNAICGKSDERDDDSKCNPQEKLTAQPCNEFLCP
Q
WKTGDWSECLVTCGKGYKHRQTWCQFGEERLDVRFCDSSKPESVQACQQQEC
AS
WQVGPWGQCTTTCGPGYQMRAVKCVVGSYGSVMDDTECNAATRPTDTQDCGLSQCP
VTHPVAPEPKVMPHPGHKTQ
WRFGSWTQCSATCGKGTRMRYVSCRDQQG
GVAEESACAHLPKPPASEVCSIVACGQ
WKVLEWTACSVSCGQGTTTRQVVCMNISDQVVELSECDLDDKPAAEQECAMPQCP
SRSSDHGGFSPNPDFRKKTALPGRTDRNRAGRLQAQQ
WRTGPWGACSSTCAGGFQRRVVVCQDENGYPASSCDESIQPIEQRSCESGSCP
Q
WFYGSWSECSKSCGGGIKTRLVACQRPNGERFNDLSCEILDKPPDREQCNTQSCS
INPHWSTDQWSLCFASCWSLHSSHLDQVGLQISSGESGKGGDEVDRGFFKNKDMGGGPKETDW
>dAdamTS-A NP_996218.1 *Drosophila melanogaster* Adamts-A amino acid sequence
MSMPDAGSLKSPAGGQEVECHRLLSDLPAFAYPSTTLRSTTSGHSTGTNSTTVCESPGARDRTSFACSSSCVSSACS
ATASDDDEDERALERCLRGTTDDLGESGLRYSLKGPPPELYSAKKDFISKGPLLGRQLEVKKRCEWWCHKYIQKMST
HWRQNACLYACCIAFLLGMLIMFHLGLRSAHKGQEELPQSTHPLANSPPATPATLHPRRLDNDTSTDHEPPDGLDDL
DEEEHSAFVMPTKVYNYSLSEADLIYESKRNSDINSFLKESASAFAMTGTYRNM
SNEIWDPHPQYNLNVFGRQLHLVLRQDASFVHNHSMTHIRILKEGEEHPGPETEAEAEQRHLGCFYSGYVEDDPHSM
VSVSLCGGMTGYIKTSFGALLIQPVNRTSSDE
VLHRVFRKSQRNARHAVSKFELGLDDFMSKLEQVQEEEQKSKSRKLNRKKRHYADVDNQV
YTLEVLIAVDNSMKQFHGEDLQPYILILMSIVSSIFADASIGNSIRILLVRLISLPNINDQTHSSNEMLKHFCQFIN
QSGYERDTAMLITREPICGSVPGKICHMLGLAELGTVCSSSSCSIVQDTGLPTAFTMAHELGHILNMNHDDDDKCMP
YVTRQNNNKVLHIMSSVMGIHMHPWSWSKCSRHFVSEFLEKTDKSCL
ETSVGAHIPYGTERLPGEIYSLDAQCQLSFGNDFGYCPTDEECKRLWCNRTSGNSNEQCASSNLPWADGTPCGSSGH
WCQRGKCVSNKHGYGRQVNGG
WGPWTPFTPCSLTCGGGVQESRRECNQPVPENGGKYCTGSRKKYRSCNTHQCP
PGSMDPREQQCYAMNGRNMNIPGVNPDTKWVPKYEKDACKLFCRMDMKVTYFMLKSMVTDGTSCAVDSFDKCVNGIC
RPAGCDNELNSIAKLDKCGVCEGRNDTCHEVTGNLLVSNLLGL
NDGNEPNKTLYYVTRIPKGASNIIITQRGYPDQNFIVLTDDRDNELLNGKFLKTYPLKFVYAGVTMQYTGSSSVVEQ
VNTTYSWKLSRDLIVQIISLDVSPSKRQDTVLLSYSYTI
DKPPDYEAEVEIYRWEMQAPSNCDSLCEGRSHRLPACISTTQGVKVAPQFCDKSAMPKIDDRACNTDCRLNLTVTSI
SECSAACGELGTREKTYACVQTFTNMQRSNIVDMSYCKLKFDVAYHEECREG
CWVLSEWSTCSKSCGTGSQQREAHCYLHNSRVSDDLCNPRTKPHLNTLIGICNTESCP
TYTKSPNALAVSNWVIGEWGECNEWCEKTRSVSCSHPYGIGCGSRKPKDVRKCCHIKYTSDWTDCSVQCGEGVKRKK
QSCTRVYKPDVPGTRKRRVYVDESYCISRKVHRPKLRTTTKSCRINCKWNASDWRRCPADCSEEYQTRDVRCESFQG
DGVEDKHCDAKKRPSKRRICNNCVRRQSRVISQCNCEGVEKRRDFCFNSHKGRIACPTRARVERHSCTPPPHCRRRS
AIGSSISSRPRGTGVSSSRSLNSIGGSRNRGTPR
SCADLKEMHGYNKDGNYQLEVRSRMVHIYCHGMNSRTPQEYVNVDPQENYSIYYEYRTKQTNSCPPESRGHEYYNDQ
NSGRTHFRKLRLNITDLRIMDNDFKFADSRGLAQKLGSAGDCYNRIGQCPQGDFSINMKDTDFSIRPGTVWRMHGQY
SVMKRISEFDTTTQMRRGFCGGYCGGCYIAPDSGLYLDV
L
>cGON-1 NP_001255448.1 *C. elegans* Gon-1 amino acid sequence
MRSIGGSFHLLQPVVAALILLVVCLVYALQSGSGTISEFSSDVLFSRAKYSGVPVHHSRWRQDAGIHVIDSHHIVRR
DSYGRRGKRDVTSTD
RRRRLQGVARDCGHACHLRLRSDDAVYIVHLHRWNQIPDSHNKSVPHFSNSNFAPMVLYLDSEEEVRGGMSRTDPDC
IYRAHVKGVHQHSIVNLCDSEDGLYGMLALPSGIHTVEPIISGNGTEHDGASRHRQHLV

RKFDPMHFKSFDHLNSTSVNETETTVATWQDQWEDVIERKARSRRAANSWD
HYVEVLVVADTKMYEYHGRSLEDYVLTLFSTVASIYRHQSLRASINVVVVKLIVLKTENAGPRITQNAQQTLQDFCR
WQQYYNDPDDSSVQHHDVAILLTRKDICRSQGKCDTLGLAELGTMCDMQKSCAIIEDNGLSAAFTIAHELGHVFSIP
HDDERKCSTYMPVNKNNFHIMAPTLEYNTHPWSWSPCSAGMLERFLENNRGQTQCLFDQP
VERRYYEDVFVRDEPGKKYDAHQQCKFVFGPASELCPYMPTCRRLWCATFYGSQMGCRTQHMPWADGTPCDESRSMF
CHHGACVRLAPESLTKIDGQ
WGDWRSWGECSRTCGGGVQKGLRDCDSPKSENEVETLKSNVTSRPRNGGKYCVGQRERYRSCNTQECP
WDTQPYREVQCSEFNNKDIGIQGVASTNTHWVPKYANVAPNERCKLYCRLSGSAAFYLLRDKVVDGTPCDRNGDDIC
VAGACMPAGCDHQLHSTLRRDKCGVCGGDDSSC
KVVKGTFNEQGTFGYNEVMKIPAGSANIDIRQKGYNNMKEDDNYLSLRAANGEFLLNGHFQVSLARQQIAFQDTVLE
YSGSDAIIERINGTGPIRSDIYVHVLSVGSHPPDISYEYM
TAAVPNAVIRPISSALYLWRVTDTWTECDRACRGQQSQKLMCLDMSTHRQSHDRNCQNVLKPKQATRMCNIDCSTRW
ITEDVSSCSAKCGSGQKRQRVSCVKMEGDRQTPASEHLCDRNSKPSDIASCYIDCSGRK
WNYGEWTSCSETCGSNGKMHRKSYCVDDSNRRVDESLCGREQKEATERECNRIPCP
R
WVYGHWSECSRSCDGGVKMRHAQCLDAADRETHTSRCGPAQTQEHCNEHAC
T
WWQFGVWSDCSAKCGDGVQYRDANCTDRHRSVLPEHRCLKMEKIITKPCHRESCP
KYKL
GEWSQCSVSCEDGWSSRRVSCVSGNGTEVDMSLCGTASDRPASHQTCNLGTCP
F
WRNTDWSACSVSCGIGHRERTTECIYREQSVDASFCGDTKMPETSQTCHLLPC
TS
WKPSHWSPCSVTCGSGIQTRSVSCTRGSEGTIVDEYFCDRNTRPRLKKTCEKDTCD
GPRVLQKLQADVPPIR
WATGPWTACSATCGNGTQRRLLKCRDHVRDLPDEYCNHLDKEVSTRNCRLRDCS
YWKMAEWEECPATCGTHVQQSRNVTCVSAEDGGRTILKDVDCDVQKRPTSARNCRLEPCPKGEEHIGS
WIIGDWSKCSASCGGGWRRRSVSCTSSSCDETRKPKMFDKCNEELCP
PLTNNSWQISPWTHCSVSCGGGVQRRKIWCEDVLSGRKQDDIECSEIKPREQRDCEMPPCRSHYHNKTSSASMTSLS
SSNSNTTSSASASSLPILPPVVS
WQTSAWSACSAKCGRGTKRRVVECVNPSLNVTVASTECDQTKKPVEEVRCRTKHCP
R
WKTTTWSSCSVTCGRGIRRREVQCYRGRKNLVSDSECNPKTKLNSVANCFPVACP
AYRWNVTPWSKCKDECARGQKQTRRVHCISTSGKRAAPRMCELARAPTSIRECDTSNCPYE
WVPGDWQTCSKSCGEGVQTREVRCRRKINFNSTIPIIFMLEDEPAVPKEKCELFPKPNESQTCELNPCD
SEFK
WSFGPWGECSKNCGQGIRRRRVKCVANDGRRVERVKCTTKKPRRTQYCFERNC
LPS
TCQELKSQNVKAKDGNYTILLDGFTIEIYCHRMNSTIPKAYLNVNPRTNFAEVYGKKLIYPHTCPFNGDRNDSCHCS
EDGDASAGLTRFNKVRIDLLNRKFHLADYTFAKREYGVHVPYGTAGDCYSMKDCPQGIFSIDLKSAGLKLVDDLNWE
DQGHRTSSRIDRFYNNAKVIGHCGGFCGKCSPERYKGLIFEV
NTKLLNHVKNGGHIDDELDDDGFSGDMD

Supplemental Figure 3

Full agarose gel image analysis of adamts9 expression in zebrafish embryos at different development stages

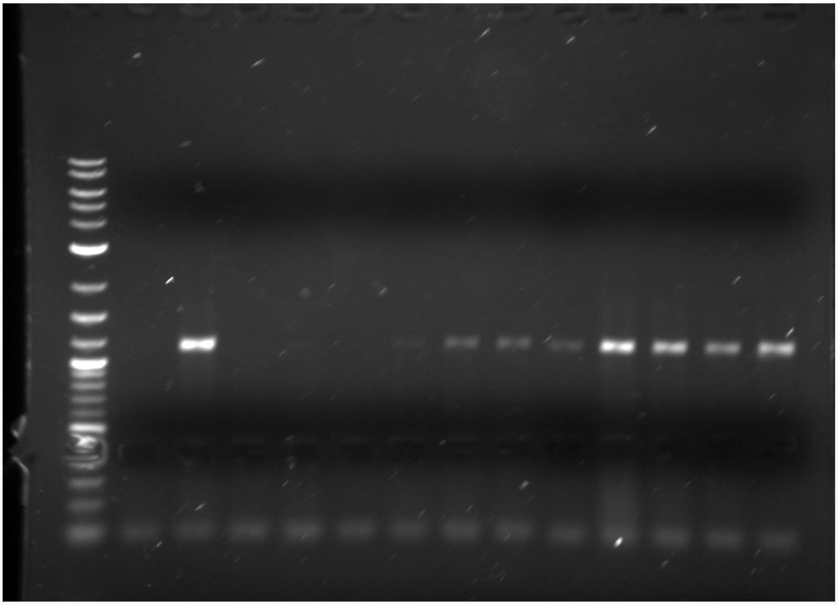

M: NEB 1kb plus DNA ladder; 1: Ovary (fully grown immature follicles); 2: Preovulatory stage IVb follicles; 3: Ovulated stage V oocytes; 4: One cell stage embryos; 5: Four cell stage embryos; 6: Oblong stage embryos (~3.5 hpf, hours post fertilization); 7: Germ-ring stage embryos (~5.5 hpf); 8: Eight somite stage embryos (~11.5 hpf); 9: 24 hpf (hours post fertilization) embryos; 10: Two dpf (days post fertilization) embryos; 11: Three dpf embryos; 12: Six dpf embryos; 13: Six wpf (weeks post fertilization) gonad.

Supplemental Figure 4

Full agarose gel image analysis of eukaryotic translation elongation factor 1 alpha 1a (eef1a1a) expression in zebrafish embryos at different development stages

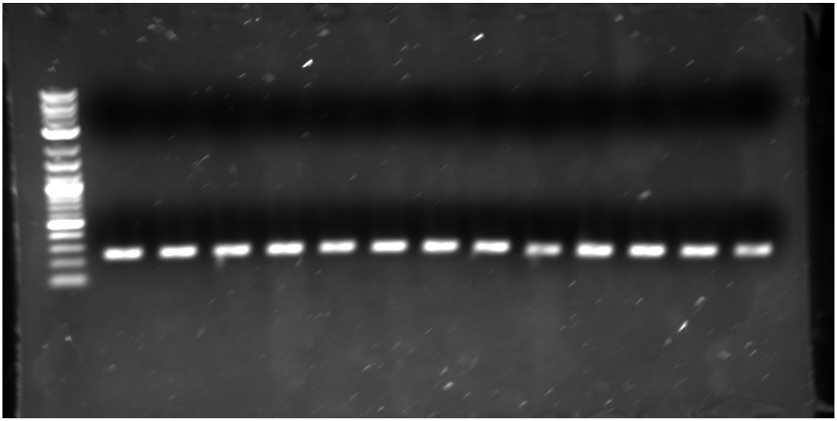

M 1 2 3 4 5 6 7 8 9 10 11 12 13

M: NEB 1kb plus DNA ladder; 1: Ovary (fully grown immature follicles); 2: Preovulatory stage IVb follicles; 3: Ovulated stage V oocytes; 4: One cell stage embryos; 5: Four cell stage embryos; 6: Oblong stage embryos (~3.5 hpf, hours post fertilization); 7: Germ-ring stage embryos (~5.5 hpf); 8: Eight somite stage embryos (~11.5 hpf); 9: 24 hpf (hours post fertilization) embryos; 10: Two dpf (days post fertilization) embryos; 11: Three dpf embryos; 12: Six dpf embryos; 13: Six wpf (weeks post fertilization) gonad.

Supplemental Table 1. Percentage of squence identities (top right) and similarities (bottom left) of Adamts9 prodomain among representative species. Zebrafish has two prodomins, in which only two amino acids were different. We used first prodomain for comparison.

	human	mice	zebrafish	*Drosophila*	*C. elegans*
human		77	51	29	24
mice	81		43	27	22
zebrafish	60	56		30	29
Drosophila	42	37	42		23
C. elegans	38	28	39	35	

Supplemental Table 2. Percentage of squence identities (top right) and similarities (bottom left) of Adamts9 metalloproteinase domain among 5 distant species.

	human	mice	zebrafish	*Drosophila*	*C. elegans*
human		88	81	45	53
mice	92		77	42	50
zebrafish	91	85		45	55
Drosophila	60	59	60		40
C. elegans	68	65	71	55	

Supplemental Table 3. Percentage of squence identities (top right) and similarities (bottom left) of Adamts9 spacer domain among five distant species.

	human	mice	zebrafish	*Drosophila*	*C. elegans*
human		78	76	31	47
mice	90		78	32	42
zebrafish	91	88		33	46
Drosophila	52	49	52		33
C. elegans	66	59	63	48	

Supplemental Table 4. Percentage of squence identities (top right) and similarities (bottom left) of Adamts9 gon-1 domain among five distant species. Zebrafish Adamts9 lacks gon-1 domain and thus was not included for comparison.

	human	mice	*Drosophila*	*C. elegans*
human		92	34	41

mice	95		35	41
Drosophila	52	50		34
C. elegans	56	56	48	

Note. EMBOSS Stetcher was used for all pairwise comparison.
